# Dynamics and ligand-induced conformational changes in human prolyl oligopeptidase analyzed by hydrogen/deuterium exchange mass spectrometry

**DOI:** 10.1038/s41598-017-02550-1

**Published:** 2017-05-26

**Authors:** Alexandra Tsirigotaki, Roos Van Elzen, Pieter Van Der Veken, Anne-Marie Lambeir, Anastassios Economou

**Affiliations:** 1 KU Leuven, Department of Microbiology and Immunology, Rega Institute, Laboratory of Molecular Bacteriology, Herestraat 49, PO Box 1037, B-3000 Leuven, Belgium; 2 Laboratory of Medical Biochemistry, Department of Pharmaceutical Sciences, University of Antwerp. Universiteitsplein 1, B-26 10 Antwerp, Belgium; 3 Laboratory of Medicinal Chemistry, Department of Pharmaceutical Sciences, University of Antwerp. Universiteitsplein 1, B-2610 Antwerp, Belgium

## Abstract

Prolyl oligopeptidase (PREP) is conserved in many organisms across life. It is involved in numerous processes including brain function and neuropathology, that require more than its strict proteolytic role. It consists of a seven-bladed β-propeller juxtaposed to a catalytic α/β-hydrolase domain. The conformational dynamics of PREP involved in domain motions and the gating mechanism that allows substrate accessibility remain elusive. Here we used Hydrogen Deuterium eXchange Mass Spectrometry (HDX-MS) to derive the first near-residue resolution analysis of global PREP dynamics in the presence or absence of inhibitor bound in the active site. Clear roles are revealed for parts that would be critical for the activation mechanism. In the free state, the inter-domain interface is loose, providing access to the catalytic site. Inhibitor binding “locks” the two domains together exploiting prominent interactions between the loop of the first β-propeller blade and its proximal helix from the α/β-hydrolase domain. Loop A, thought to drive gating, is partially stabilized but remains flexible and dynamic. These findings provide a conformational guide for further dissection of the gating mechanism of PREP, that would impact drug development. Moreover, they offer a structural framework against which to study proteolysis-independent interactions with disordered proteins like α-synuclein involved in neurodegenerative disease.

## Introduction

Prolyl oligopeptidase (PREP, EC 3.4.21.26) is a proline-specific serine endopeptidase, present in many organisms from all kingdoms of life^1^. In humans, even though it is present in many different cell types, current investigations are highly focused on the roles of PREP in the brain^2, 3^. In addition to its enzymatic function, these studies and others support the hypothesis that PREP might be involved in neurogenesis, hippocampal plasticity and spatial memory formation both in healthy and diseased states^2, 3^. Protein-protein interactions rather than proteolytic activity seem to underlie the actions of PREP in synaptic plasticity^4–6^. For example, PREP^−/−^ mice have growth cone formation defects that can be rescued in cell culture by transfection with a gene encoding PREP or a mutant lacking proteolytic activity. Moreover, PREP affects the aggregation and clearance of α-synuclein, which itself is not cleaved by PREP^4–6^. The fact that inhibitors directed against the active site have an effect on the non-peptidase actions of PREP can be explained by a dynamic structural heterogeneity of PREP or conformational changes induced by ligand binding. Therefore, despite the dearth of mechanistic insight in its non-peptidase function, the two processes appear to be conformationally connected.

**Figure 1:**
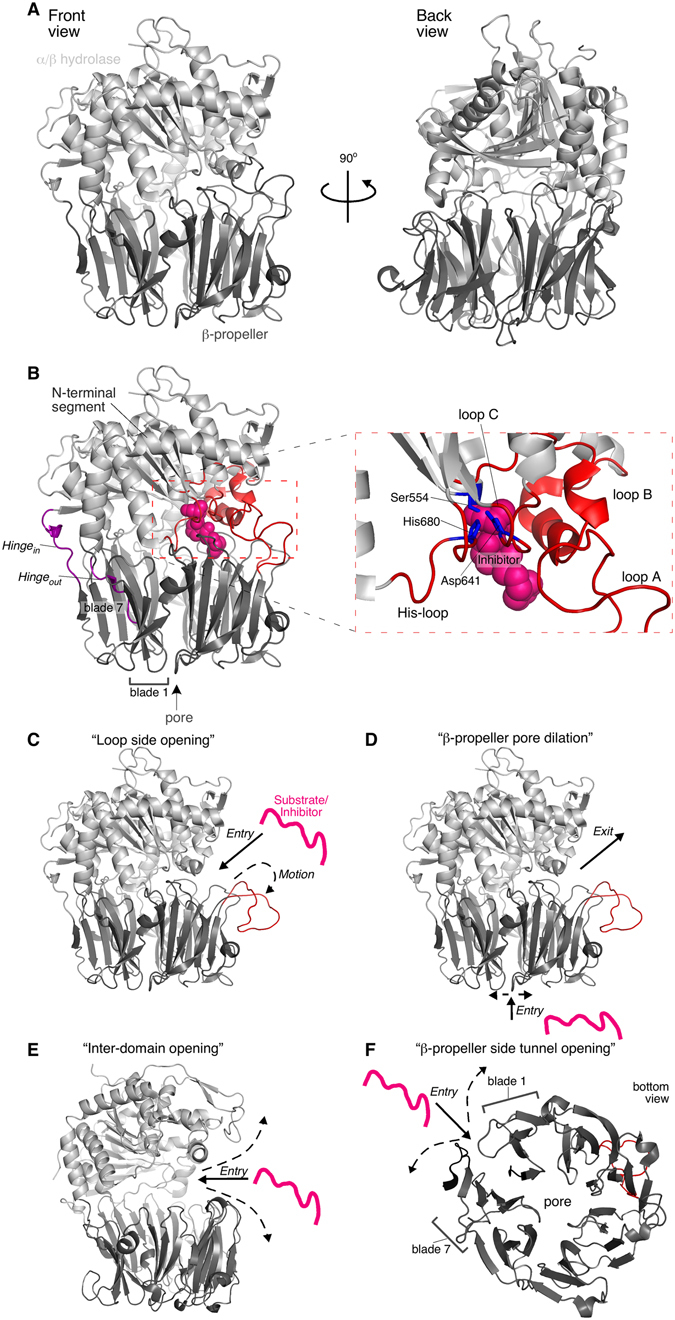
Structure of PREP and current models for the substrate gating and molecular function mechanisms. (**A**) PREP structure and domain organization (PDB accession entry: 1H2W) in a front (left) and back (right) view. Human PREP and its homologues are two-domain assemblies consisting of an α/β hydrolase domain (light grey) and a 7-bladed β-propeller (dark grey). (**B)** The two domains are connected through a two-linker hinge (purple). *Hinge*_*out*_ connects the N-terminal segment of the α/β hydrolase domain with the first β-propeller β-strand. *Hinge*_*in*_ links the last β-propeller strand with the rest α/β hydrolase domain. The catalytic triad in the α/β hydrolase domain comprises of the His680 residue from the His-loop (residues 676–685), Asp641 from loop C (636–646) and Ser554 (right; zoom in view of the active site and its surrounding loops; catalytic triad in blue; loops in red). PREP substrates and its hydrolase inhibitors bind to the active site (PDB accession entry 4AN0; inhibitor KYP-2047 shown as pink spheres). Loops A (189–209) and B (577–608) (red) surround the active site. The propeller-like configuration of the β-blades in the β-propeller domain create a ~4Å-wide pore. The blade unit is 4 antiparallel β-strands. (**C–F)** Current models for the substrate gating mechanism of PREP. A hypothetical substrate/inhibitor is drawn as a pink line; dash-lined arrows represent motions of PREP regions related to gating; continuous-lined arrows indicate the suggested substrate entry pathway. (**C)** Loop A (red) has been suggested to move outwards enabling side entry of PREP substrates (PDB accession entry: 1H2W; loop A was manually translated outwards using PyMOL Molecular Graphics System, Version 1.8 Schrödinger, LLC). (**D)** The β-propeller pore may dilate for substrate encapsulation, while loop A outward motions may provide the exit pathway (structure as in panel C). (**E)** Bilateral opening of the inter-domain interface by large scale outward motions of both domains around the hinge would expose the active site (PDB accession entry: 4BP8; *Trypanosoma brucei* Prolyl Oligopeptidase B). (**F)** The unclosed β-propeller between blades 1 and 7 may provide a side tunnel by weakening interactions between the two β-stranded blades (bottom view of the β-propeller; structure as in panel A).

The structure of PREP is characteristic of the prolyl oligopeptidase family (S9)^7^. It consists of two domains (Fig. 1A): a discontinuous α/β-hydrolase domain (1–71 and 428–710, human PREP numbering) that contains the catalytic triad (Ser554, His680, Asp641; Fig. 1B, right) and a juxtaposed seven-bladed β-propeller (72–427). The two domains are covalently connected only from the one side of PREP with a two-linker hinge (*hinge*_*out*_: residues 72–79; *hinge*_*in*_ residues 424–434; Fig. 1B). All mammalian PREP structures determined so far, in the free or inhibitor-bound states, are in a closed conformation in which the catalytic triad and the inhibitor/substrate binding site are buried in the inter-domain interface, surrounded by an extended network of hydrophobic contacts, hydrogen bonds and salt bridges between loops and turns from both domains. In this closed state PREP has a fairly substantial internal cavity that connects to external solvent by a narrow pore (~4 Å) in the β-propeller domain core^7–9^, of insufficient width for substrate entry.

Based on several different structures of the *Aeromonas punctata* PREP, an induced fit mechanism was proposed where PREP is in an open conformation, exposing the internal cavity to the solvent, and the catalytic site is assembled upon substrate binding leading to a closed conformation similar to the ones previously determined^10^. On the other hand, there is experimental evidence that mammalian free PREP is distributed between different conformations and that ligand binding shifts this equilibrium to a single state, i.e. conformational selection^11, 12^. Both induced fit and conformational selection are consistent with substrate hydrolysis kinetic studies of PREP, showing that the experimental kinetic parameters are substrate-dependent and that a physical rather than a chemical step is rate determining^13^. Since functionally essential residues in the substrate binding pocket and the inter-domain interface are conserved between species, domain movement may be common in the catalytic cycle of all PREPs^14^.

Despite the evidence for the structural heterogeneity of PREP and conformational changes induced by ligand binding, it has been challenging to model these changes using computational methods^10, 11, 13–22^. Molecular dynamics simulations suggest that ligands access the active site from the open side by rather limited rearrangements of the loops covering the ligand binding site (Fig. 1B) without significant disruption of inter-domain interactions^15, 17, 23^. Specifically, outward motion and detachment of loop A (189–209) from loop B (577–608) (Fig. 1C; “Loop side opening”), with concomitant disruption of loop B and C (636–646) interactions, may be a possible gating mechanism^17^. However, using combined computational methods, substrate entry through the β-propeller pore and product exit from the side opening were also proposed (Fig. 1D; “β-propeller dilation”)^24^. Moreover, molecular dynamics and normal mode analysis of concerted long range dynamics suggested that the natural motions of PREP would allow for bilateral opening of the two domains around the hinge by loosening of the inter-domain interactions (Fig. 1E; “Inter-domain opening”, otherwise known as “Pacman” model), access of substrate by dilation of the β-propeller core or by widening the side opening, and rearrangement of the inter-domain loops at the side opening^14^. While the extent of inter-domain opening in the mammalian PREP is not known, crystal structures of the bacterial PREP at the free state show varying inter-domain distances^10^ at an asymmetric opening^10, 25^, at which the back face retains inter-domain contacts^10, 25^. Additionally, the atypical β-propeller of PREP, where blades 1 and 7 are not fully closed, in contrast to a “Velcro-like” closing common in other β-propellers of this class, raised the possibility that separation of blades 1 and 7 may provide an alternative side tunnel^8^ (Fig. 1F; “β-propeller side tunnel opening”). Immobilization of the two blades by engineered disulfide cross-linking, has been suggested to prevent substrate binding to the catalytic site^8^.

Despite the significant insights provided by computational analysis, the conformational dynamics of PREP that are involved in the gating mechanism remain elusive. Hydrogen Deuterium eXchange followed by Mass Spectrometry (HDX-MS) is a powerful structural biology tool to probe protein-ligand interactions and conformational dynamics at near-residue resolution^26, 27^. The method is based on the natural hydrogen exchange of solvent-accessible, non-hydrogen bonded backbone amides with the solvent. These breathing motions are “captured” by deuterium labeling from the solvent. Here we probed the conformational dynamics of human PREP with and without an inhibitor bound in the active site by HDX-MS. We demonstrate significant changes in the dynamics of PREP between the two states at high resolution. Inhibitor binding stabilizes the previously labile front inter-domain interface and the α/β-hydrolase domain helix of the His-loop becomes ordered. This conformational signal is transmitted to the adjacent β-propeller blade 1 loop. This may lock the inter-domain interface at this site, and stabilizes the hinge. These findings corroborate only some of the existing gating models and reveal new regulatory elements, facilitating future dissection of the gating mechanism of PREP.

## Results

### HDX-MS of PREP in the free state

PREP may exist in equilibrium between open and closed states related to its gating mechanism^12, 19^. Native gel electrophoresis revealed that the free enzyme populates at least two conformational species that migrate distinctly (Fig. S1A, left). To follow the dynamics of its conformational flexibility in solution at higher resolution we employed HDX-MS.

Backbone amide hydrogens (NHs) from all amino acids, except for the imino acid proline, participate in hydrogen bonds for secondary and tertiary structure. Structural fluctuations interrupt these local interactions and may render the NHs solvent-accessible and competent for exchange with D from the solvent (D_2_O) used in HDX-MS^28^. Replacement of H for D labels NHs and “captures” these fluctuations. In globular proteins, non-hydrogen-bonded, surface-exposed, unstructured regions are in rapid H/D exchange. Structured regions exhibit H/D exchange rates spanning over 100 orders of magnitude, depending on the fractional occupancy of the hydrogen bond, solvent accessibility and protein dynamics for local or large-scale fluctuations. D incorporation is determined after proteolysis, by mass spectrometry, due to the ~1 Da difference between ^1^H and D, to a spatial resolution of ~5 residues^29, 30^.

**Figure 2:**
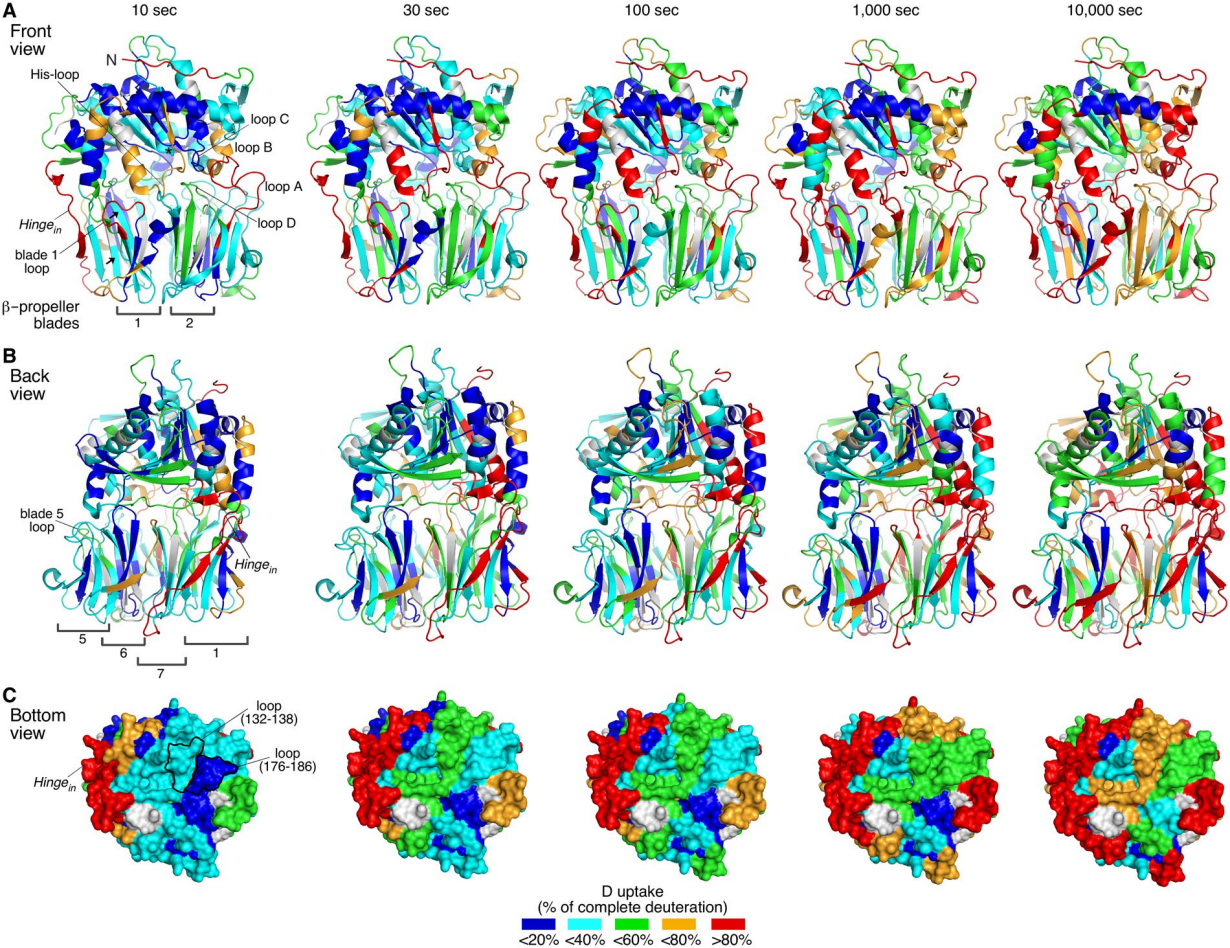
Conformational dynamics of PREP at the free state by HDX-MS. (**A–C)** Deuterium (**D**) uptake profiles of substrate/inhibitor-free PREP as a function of incubation time in deuterated buffer, presented in a front (**A**) and back view (**B**) of the inter-domain interface and in a bottom view of the β-propeller domain (**C**; surface representation; pore view) (detailed data in Fig. S2 and Table S1). D uptake values are expressed as a percentage relative to the complete deuteration control and are mapped on the porcine PREP structure (97.1% primary structure identity with human PREP), crystallographically resolved at the free state, using a color gradient, as indicated (bottom; grey: not identified regions). Important structural elements and peptides discussed in the text are indicated. Only some blades of the β-propeller are enumerated for simplicity. Inter-domain loops are indicated by the number of the blade in which they reside. Arrows indicate the β1 and 2 strands of blade 7. The asterisk indicates the Ser554 residue of the active site. *n* = *3*.

PREP was isotopically labeled (pD 7.4, T = 25 °C) for 10, 30, 100, 1,000 and 10,000 sec, digested with pepsin and peptic fragments covering 91.8% of the sequence were identified by MS (Fig. S2). The fractional D uptake, relative to the maximum measured D incorporation, for each peptide was mapped on the crystal structure using a color gradient (Fig. 2A–C; hot/cold colors indicate high/low D uptake, respectively). In the absence of available structure of human PREP in the free state, that of the porcine homologue was used. The two proteins are 97.1% identical and structurally superimpose (RMSD = 0.225 Å; PDB accession numbers 3DDU and 4AN0).

At the shortest HDX pulse (i.e. 10 sec) regions that have complete or almost complete H/D exchange comprise surface exposed loops and β-strands (>80% D uptake; Fig. 2A–C; red). These regions include the extreme N-terminal unstructured peptide, the loop connecting the α-helices of the N-terminal α/β hydrolase segment and the inter-domain *hinge*_*in*_. Peptides corresponding to α-helices and β-strands are significantly protected (blue, cyan). These data are in general agreement with the crystal structure^7, 8^.

### A rigid β-propeller domain with two flexible blades in the free state of PREP

The β-propeller is rather rigid (Fig. 2B–D), corroborating molecular dynamics simulations^17^. Each blade contains four anti-parallel β-strands, with each blade pair positioned face-to-face. This maximizes their interaction network through hydrogen bonds, hydrophobic contacts and salt bridges, with strong involvement of intra- and inter-blade connecting loops. This is reflected in the low D uptake of both the β-strands and most of the loops of the β-propeller (Fig. 2A–C). The loops at the entrance of the central β-propeller pore do not exhibit significant dynamics and appear quite stabilized, e.g. the blade 3 loop facing inwards the pore (Fig. 2C; peptide 176–186).

Despite the overall rigidity of the β-propeller domain, the dynamics of blades 2 and 7 are the fastest compared to those of the other blades (Fig. 2A,B). Specifically, blade 2 (peptides 128–138; β1, 139–149; β2, and 156–168; β4, the latter with slightly slower kinetics of exchange) is located in the immediate proximity of loop A and the His-loop and exhibits moderate D uptake already at 10 sec. The peptide covering β1 of blade 2 has 50% loop content which could account for its relatively fast H/D exchange kinetics. However, its fast kinetics are attributed to β1, based on the slower kinetics of its overlapping peptide 132–138 (Fig. S2), containing mainly loop and only one β-strand residue. Apparently, the blade 2 β-strands undergo opening transitions. The absence of overlapping peptides for the blade 7 β-strands 1 and 2 (Fig. 2A; arrows) prevents a similar analysis on the β-strands *per se*. However, the significant protection of the succeeding β3 strand of blade 7 (peptide 402–412) indicates that at least β3, the direct interactor of β2, is stable. The apparent instability of blade 2, and potentially of blade 7, observed here may explain why the β-propeller is slightly destabilized when bound to the α/β hydrolase domain^16^.

### A rigid inner core of the α/β hydrolase domain in the free state of PREP

**Figure 3:**
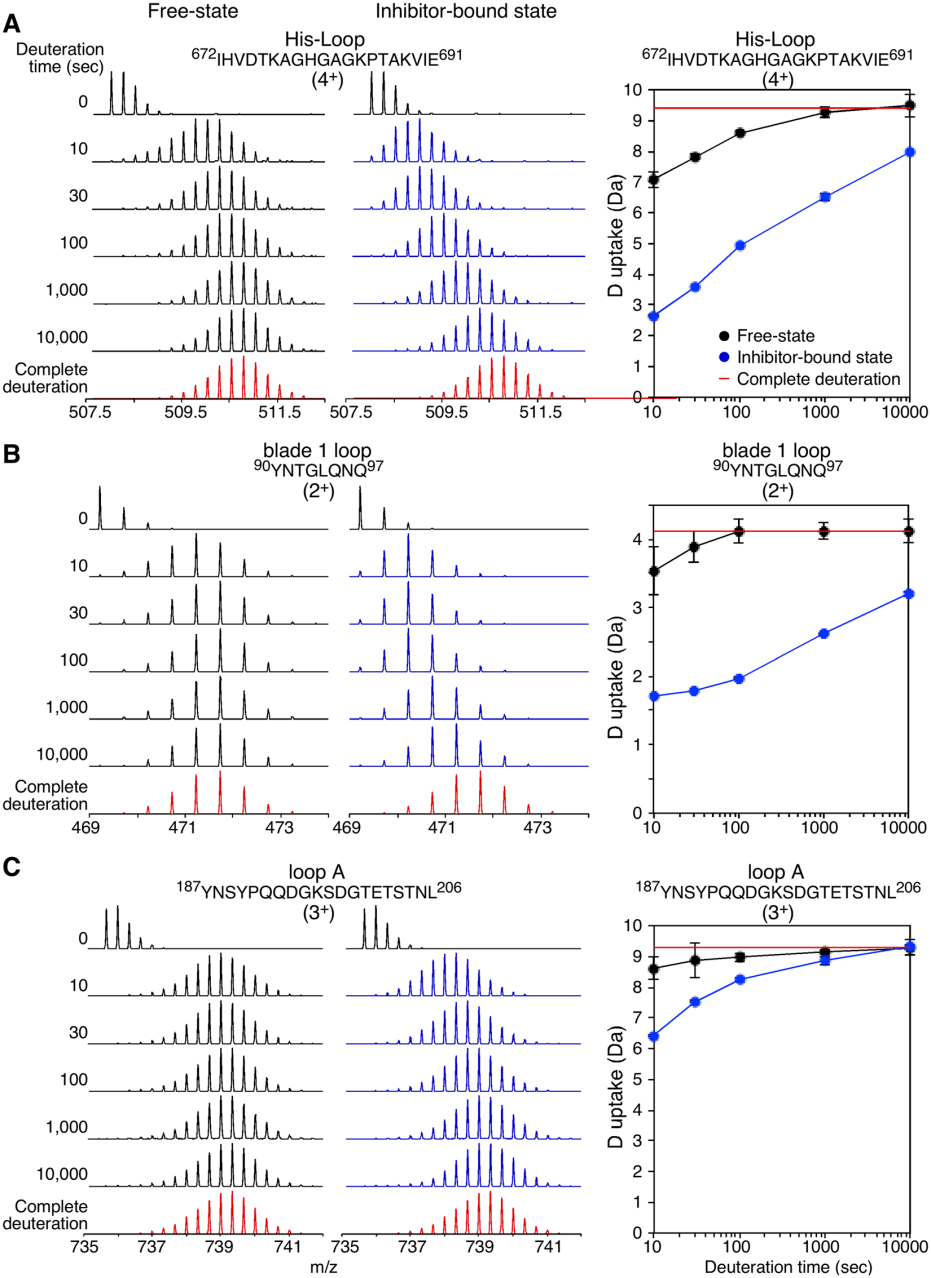
Representative HDX-MS spectra of PREP at the free and inhibitor-bound states. (**A–C)** Mass spectra (left) and deuterium (**D**) uptake plots (right) of representative peptides covering the regions of the His-loop and its succeding α-helix (**A**), the β-propeller loop at the inter-domain interface that connects β2 and 3 of blade 1 (**B**; blade 1 loop) and of loop A (**C**) are shown at the indicated H/D exchange time course (top, left) at the free (black) and inhibitor-bound (blue) states. The complete deuteration control is highlighted in red. Amino acid sequences and charge states are indicated. *n = 3*; Standard Deviations (SD) are shown in the D uptake plots.

The inner core of the α/β hydrolase domain, including the catalytic site (Ser554; Fig. 2A, asterisk; peptide 536–559), remains substantially rigid over time. Increased flexibility is observed at the turns and helical edges of the N-terminal segment (peptide 30–41), suggestive of helix-coil transitions, typical for helical edges^31^. The His-loop (Fig. 2A; peptide 672–691), part of the catalytic triad (Fig. 1B; right), and loop B (Fig. 2A; peptides 584–594, 584–602), surrounding the substrate-binding site (Fig. 1B), have loose structure and/or fast dissociation of hydrogen bonds with neighboring interacting regions (Fig. 2A; >60% D uptake). The His-loop peptide 672–691 also covers two turns of its succeeding α-helix. The increased dynamics of this region (Figs 2A and 3A; black) suggest that the helical segment is not very rigid.

### The inter-domain interface contains flexible loops in the front side of PREP

The two sides of the α/β hydrolase/β-propeller interface exhibit distinct conformational dynamics (Fig. 2A and B). Regions with increased flexibility are clustered in the front surface of PREP (Fig. 2A; >60% D uptake). In the β-propeller, these are the blade 1 loop (peptide 90–97), and loop A (187–206) (Fig. 3B and C respectively; black, and Fig. 2A). In the α/β hydrolase domain, loop B (peptides 584–594, 584–602), the His-loop and its subsequent α-helix (peptide 672–691) contribute to the fast conformational dynamics of the front inter-domain region (Figs 2A and 3A; black).

While front loops take up D readily, almost half of the loops at the back side of the interdomain interface, opposite to the His-loop, have low D uptake at short labeling pulses (<1 min) (Fig. 2B). At least two of them, stemming from β-propeller blades 5 and 6, remain significantly rigid over the time course kinetics, indicating restricted flexibility.

Overall, H/D exchange suggests that while the α/β hydrolase and β-propeller domains of PREP are substantially rigid, the inter-domain interface at its front is significantly dynamic.

### Inhibitor binding induces structural rigidity in PREP

Several PREP inhibitors, like the prototypical KYP-2047, are available that covalently interact with the catalytic serine (Fig. 1B, right; Fig. S1B)^32^. KYP-2047 is a reversible competitive inhibitor with a sub-nanomolar inhibition constant that covalently interacts with the catalytic Ser554^17^. KYP-2047 is best described as a slow tight-binding inhibitor with an on-rate constant of 1.75 × 10^6^ M^−1^s^−1^ at 25 °C^33^. Binding of KYP-2047 is complete by ~80 sec (Fig. S1C) and causes important structural changes that reduce the various conformational states or PREP to a single species (Fig. S1A, right). It also reduces the number of PREP cysteinyl residues that are accessible to an externally added probe (Fig. S1D).

**Figure 4:**
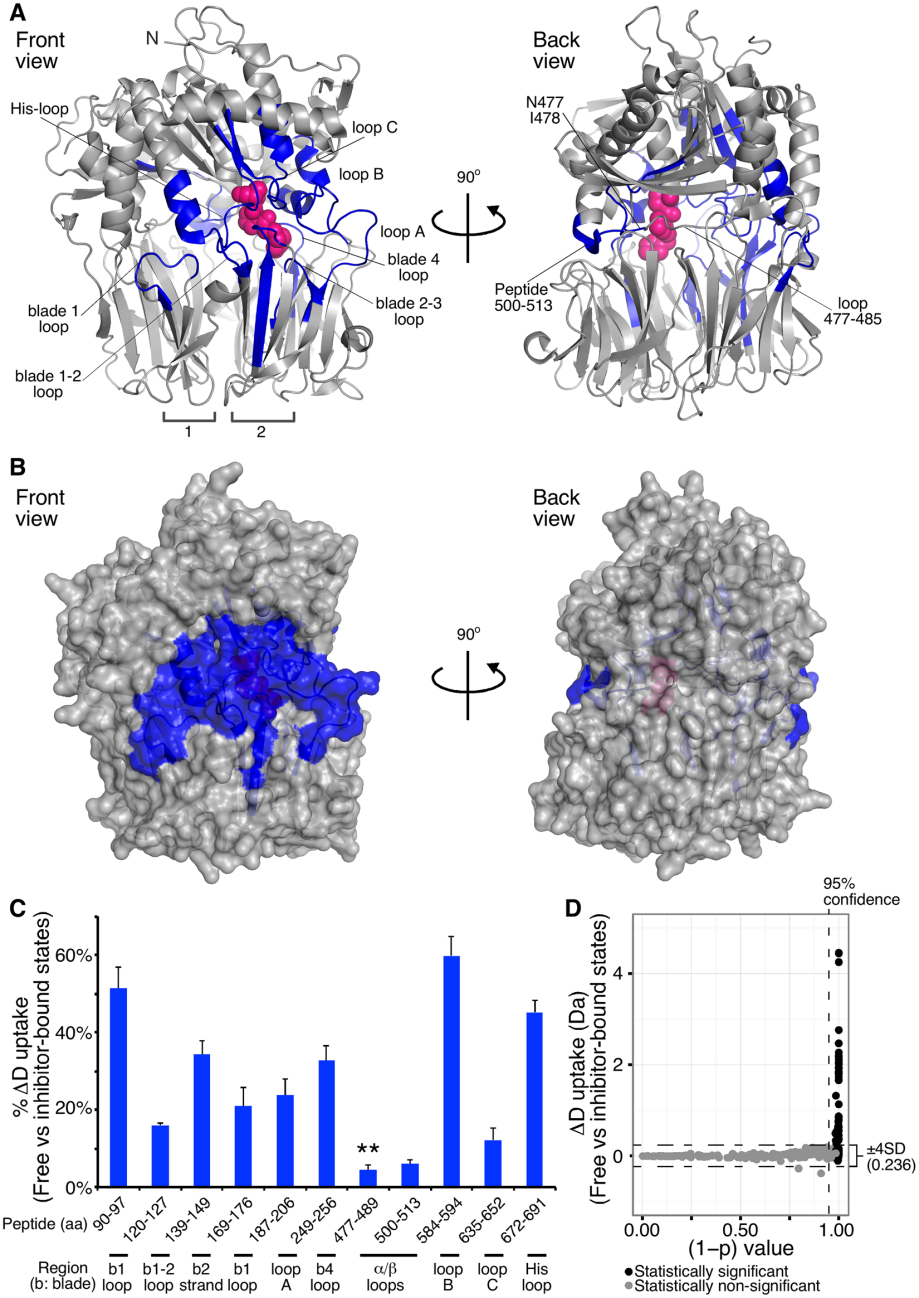
Fast change in the dynamics of PREP in response to inhibitor binding probed by comparative HDX-MS. (**A,B)** Map of the human PREP regions that show statistically significant reduced D uptake at <1 min of incubation in deuterated buffer upon inhibitor binding. (blue: protected regions indicated correspondingly; loops are represented by the number of the blade in which they reside; inter-blade connecting loops are separated with dash; numerical values and statistical analysis in Table S1). *n* = *3*. (**A**) Ribbon representation of the porcine PREP structure bound to the KYP-2047 inhibitor (pink spheres; Fig. S1B) from a front (left) and back (right) view of the inter-domain interface (PDB accession entry: 4AN0; where KYP-2047 is labeled IC-3). (**B)** Surface representaion of the porcine PREP structure bound to the inhibitor from a front (left) and back (right) view, as in panel A. The indicated loop 477–485 was found by overlapping peptide analysis to be protected only at the residues N477, I478 (highlighted in blue). (**C)** % D uptake difference in the free versus the inhibitor-bound state (t < 1 min) expressed as the ratio of the absolute D uptake difference between the two states to the maximum experimentally determined D uptake for a specific peptide. The maximum difference within 1 min labeling is given. The structural region of each peptide is indicated (bottom; b indicates blade). **Protection of the indicated peptide was attributed to residues N477, I478 (see Table S1 and Fig. S3). Peptide 139–149 includes loop D. *n = 3*; Standard Deviations (SD) are shown. (**D)** Scatter plot visualization of the statistical significance of the D uptake differences shown in panels A–C. All identified PREP peptides at 10 and 30 sec of isotope labeling were included. The 95% confidence threshold is indicated on the y axis; the ± 4 SD cut-off (value given on the right) is indicated by dashed lines on the x axis; black spheres indicate peptides for which the D uptake difference exceeds twice the sum of the SDs of each state for the given peptide. Black spheres above the two indicated thresholds are considered as statistically significant different peptides between the two states.

**Figure 5:**
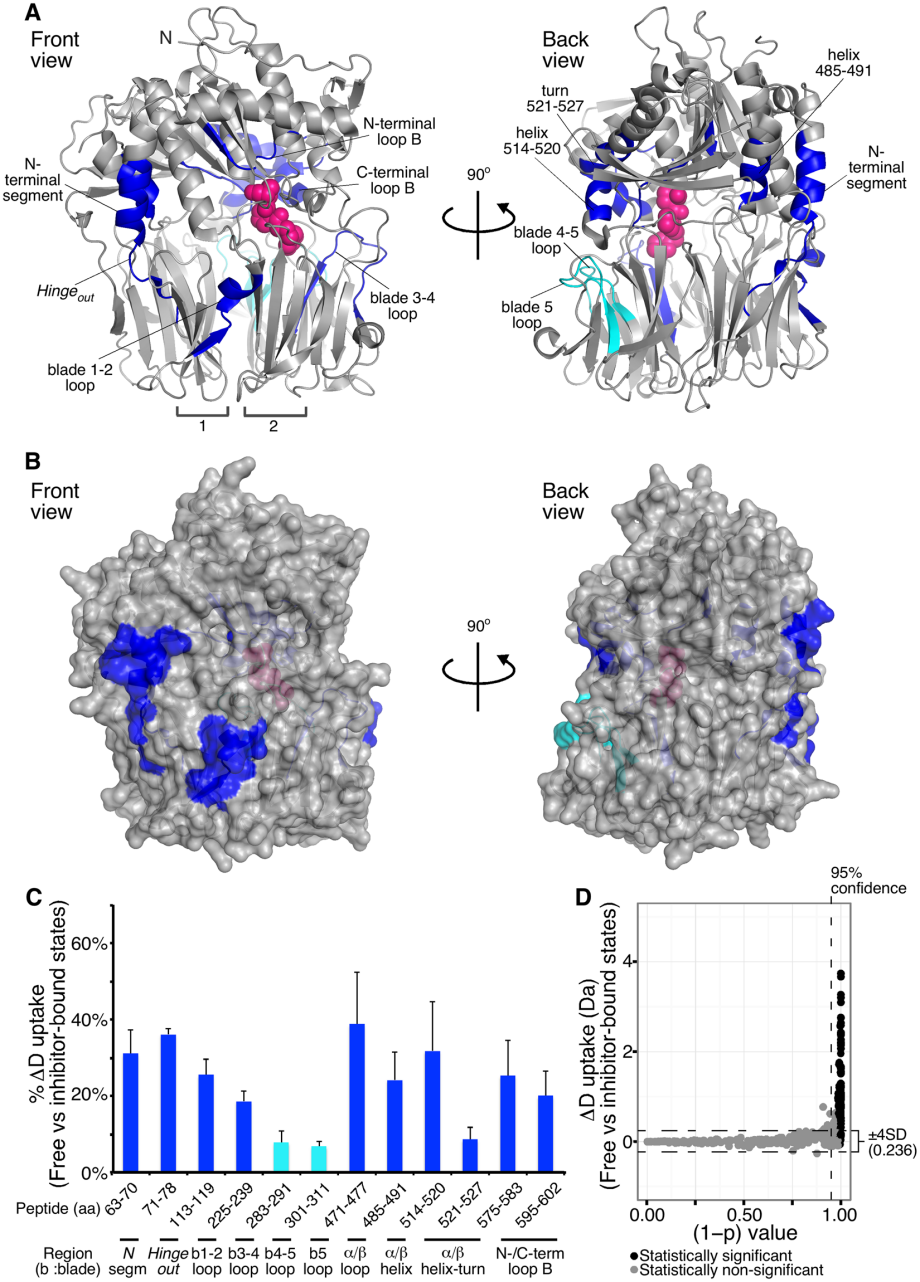
Delayed change in PREP dynamics in response to inhibitor binding probed by comparative HDX-MS. (**A,B)** Map of the PREP regions that show protection towards H/D exchange at ≥100 sec of incubation in deuterated buffer upon inhibitor binding (as in Fig. 4; numerical values and statistical analysis in Table S1). Regions with differences are highlighted in blue and indicated (loops are assigned by the number of the blade in which they reside; inter-blade connecting loops are separated with dash). Cyan: difference of <10% and observed only at one intermediate time point. (**C)** % Difference in deuterium (D) uptake of human PREP in the free versus the inhibitor-bound state (as in Fig. 4) at isotope labeling times of ≥100 sec where the maximum difference is observed (Table S1). Colors as in panel A. The structural region of each peptide is indicated (bottom; b indicates blade; N segm indicates N-terminal segment). *n = 3* for each state; Standard Deviations (SD) are shown. (**D)** Scatter plot visualization of the statistical significance of the differences in D uptake between the free and inhibitor-bound PREP states shown in panels A–C (as in Fig. 4). All human PREP identified peptides at ≥100 sec of isotope labeling were included.

To localize and identify the structural stabilization changes induced by the inhibitor, we performed comparative HDX-MS analysis at the free and inhibitor-bound states of PREP (Figs 3–5 and Table S1). For optimal visualization and interpretation of these results, we divided the inhibitor effect on PREP dynamics in two classes of stabilization effects: fast (<1 min; Fig. 4) and slow (≥100 sec; Fig. 5). The first class includes regions that either directly interact with the inhibitor, or that are affected indirectly, but strongly, in their conformations by inhibitor binding. In the second class (slow), inhibitor binding stabilizes pre-existing hydrogen bonds with moderate turnover rates in the free state and includes two types of regions: those that are conformationally affected only indirectly, and those that are interfacing with the inhibitor, but are significantly stable in the free state. The late response of inhibitor-interfacing regions could also be attributed to non-simultaneous and tight inhibitor binding by all residues or to uneven translation of the conformational cascade during allosteric regulation.

### Rapid conformational response of PREP to inhibitor binding

Regions with fast protection in the inhibitor-bound state (Fig. 4) are mainly the most flexible ones in the free state (Fig. 2B) and are located at the front inter-domain interface (Fig. 4A and B). The differences shown at 10 sec for the steady state (pre-incubation with the inhibitor) were identical with those observed when the inhibitor was added during isotope labeling (Fig. S4) indicating fast inhibitor binding at the saturating conditions used and rapid conformational response of PREP.

Regions protected from D exchange that are known from the crystal structure to be in direct contact or within <5 Å of the inhibitor are (Figs 4A and C and S5): loop C, the His-loop, the inter-domain loop connecting blades 2–3 located underneath loop A, loop B and its neighboring blade 4 loop from the β-propeller (peptide 249–256), and the loop 477–485 of the hydrolase domain. The loop 477–485 is located inside the inter-domain cavity and spans from the active site towards the hinge region. The combination of solvent inaccessibility due to the proximal inhibitor and induced conformational rigidity could explain the protection of these regions. This is more evident in the His-loop and loop B that were significantly flexible in the free state (Fig. 2B) and become strongly protected (Fig. 4C), while the more stable loop C (Fig. 2A) is less conformationally affected by inhibitor (Fig. 4C). The significantly reduced D uptake of the His-loop peptide (Figs 3A, blue and 4C) suggests inhibitor-induced stabilization of its helical segment.

Specifically for loop 477–485, the acquisition of several overlapping peptides with different D uptake profiles (Table S1, Figs S2 and S3) allowed us to localize the early stabilization effect to Ile478, and Asn477 (see discussion in Fig. S3). In contrast, the long loop at the inter-domain interface (residues 479–484) is unaffected by the inhibitor throughout the H/D exchange time course (Table S1).

The stabilization of the inhibitor-interfacing regions is transmitted to blade 1 and blade 1–2 loops (peptides 90–97 and 120–127, respectively), loop A, the flexible strand of blade 2 and its succeeding loop that do not directly contact the inhibitor. This conformational rigidity is allosteric in nature and could be rationalized by the extended network of hydrogen bonds (Fig. S6) and other interactions between these regions and the inhibitor-interactors as evidenced by the crystal structure. For instance, the blade 1 loop directly interacts with the helical segment of His-loop through at least two backbone hydrogen bonds (Fig. S6A), as well as with the blade 1–2 loop (Fig. S6B). In addition, the blade 1–2 loop forms hydrogen bonds with loop D (residues 145–151) that is directly interacting with both the inhibitor and loop C at the inter-domain interface. Loop A is hydrogen-bonded with the inhibitor-interacting loop B and the blade 2–3 loop (Fig. S6). Also, loop 500–513 of the hydrolase domain, proximal to the B and 477–485 loops, becomes indirectly slightly protected.

The observed differences in the free and inhibitor-bound states were statistically significant (Fig. 4D) and were confirmed with overlapping peptides, where available (Table S1). The degree of protection increases over time (Fig. S3 and Table S1) for the blade 1 loop (up to 52% protection), the blade 4 loop (up to 36%), the loop 500–513 (up to 15%) and the loops B and C (up to 64 and 52% respectively), suggestive of further stabilization of existing hydrogen bonds, while it decreases for the flexible His-loop and loop A.

### Enhanced stability in the slow dynamics of PREP by inhibitor binding

The induced rigidity on the flexible loops of PREP by inhibitor-binding is likely relayed to some of the regions shown in Fig. 5, for instance to the *hinge*_*out*_ loop (peptide 71–78), in an allosteric manner that stabilizes existing interactions. Stabilization of the second helix of the N-terminal α/β hydrolase segment (peptide 63–70) follows (Fig. 5, Table S1), as well as of its adjacent helix with residues 485–491, suggestive of re-enforcement of their interaction or internal stability of these helices. The inter-blade 1–2 loop (peptide 113–119) (Fig. 5A), residing in the direct vicinity of the blade 1 loop, also becomes stabilized by the inhibitor.

At the inhibitor interface, four regions appear to have a late response to inhibitor binding. Specifically, the stabilization of the C-terminal segment of loop B (peptide 595–602), as well as its N-terminal loop (peptide 575–583) and the loop 471–477 of the hydrolase domain that surround the inhibitor (see Fig. S5), evolves only at long deuteration periods (t > 1,000 sec; Table S1 and Fig. 5). Similarly, the blade 3–4 loop (peptide 225–239), underneath loop B, that contains the inhibitor-interfacing Met235 has a delayed stabilization response (Fig. 5, Table S1). Slow change in dynamics is also observed in the helix-turn-helix within residues 514–527 that shields the loop 471–477 externally and is adjacent to the C-terminal segment of loop B.

Two peptides (Fig. 5; cyan) exhibited slight protection only at a single deuteration pulse in intermediate H/D exchange times (Table S1). We consider these peptides as either outliers or only slightly affected by the inhibitor. The extent of protection for these peptides is possibly small enough to be detected at other deuteration pulses, and therefore the observed difference at a single pulse may comprise the maximal inhibitor-induced protection.

In summary, all the inhibitor-interfacing residues identified by the crystal structure, except from Ser554 and Asn555 that were not compared due to covalent interaction of Ser554 with the inhibitor, are found in peptides that exhibit significantly reduced H/D exchange in the presence of inhibitor. All the inter-domain H-bonds that involve backbone amides (Fig. S6) reside in segments that were rapidly protected from D uptake (t < 1 min; Fig. 4). These are clustered in one side of the PREP interface, spanning from the blade 1 loop of the β-propeller and the His-loop of the α/β hydrolase domain to the α/β hydrolase helix-turn-helix containing Glu512 (Figs 4 and S5). The observed differences also agree with indole and methyl-labeled NMR studies, respectively showing that the inhibitor strongly affects Trp514 and methioninyl residues 235, 581 and 583^11, 12^, though HDX-MS provided a complete view of the inhibitor effect.

## Discussion

HDX-MS provided for the first time the global in-solution dynamics of human PREP in the free and inhibitor-bound states. The rapid inhibitor binding during the simultaneous addition of inhibitor and monitoring of H/D exchange (compare Fig. S4A,B) does not allow us to analyze the early steps of the entry pathway that the substrate might follow. Nevertheless, it allows the investigation of conformationally altered regions in the closed inhibited state. The combined dynamics of the free and inhibitor-bound PREP enables the testing of existing models with implications for the gating mechanism and function of PREP.

NMR and SAXS experiments have proposed that the free enzyme populates an open and closed conformation (approximately 55% open, 45% closed), an equilibrium that is shifted towards the closed conformation when inhibitor is bound^12^. While the closed state is well understood and structurally resolved, the open conformation is elusive. HDX-MS indicated that large inter- or intra-domain motions, such as complete inter-domain opening (Fig. 1E) or twisting^14^, are unlikely in the free enzyme (Fig. 2). Widespread fast dynamics, expected for major conformational rearrangements, were not observed.

Free PREP dynamics would be compatible with slight rearrangement of loops A, B, D and His that surround the substrate/inhibitor binding site. This would not require large-scale inter-domain opening, but, at least partial, disruption of several inter-domain interactions, e.g. the His-loop – blade 1 loop contacts. The back face of the inter-domain interface may not participate in the opening mechanism as suggested by the moderate dynamics and/or lack of inhibitor binding effects of inter-domain regions at blades 5, 6 and 7 (Figs 2B, 4 and 5). The hinge is also not directly involved in an opening-closing transition along its two loops, with *hinge*_*out*_ being stabilized probably as a consequence of inhibitor-induced stability of its neighboring flexible loops (Fig. 5; Fig. S3; see below). These properties and how they change upon inhibitor binding allow us to critically think of possible substrate gating mechanisms.

A “β-propeller pore dilation” model (Fig. 1D) is less favored by PREP dynamics (Fig. 2). Pore dilation would have disrupted at least inter-blade interactions of the β-propeller, concomitantly leading to disrupted H-bonds in the outer β-propeller loops that form the central pore. However, β-propeller blades (Fig. 2A and B) and central pore loops (Fig. 2C; e.g. loop 176–186) appear substantially rigid in the free enzyme, in agreement with computational^15, 17, 23^ and denaturation studies^16^, and unaffected by inhibitor binding (Figs 4 and 5).

The “β-propeller side tunnel opening” model (Fig. 1F)^8^ is also less likely (Figs 4 and 5)^16^. Blade 1 β-strands are not significantly dynamic (Fig. 2A,B), and none of the blade 1 and 7 β-strands are inhibitor-affected (Figs 4 and 5), that would corroborate blade 1,7 separation. In addition, *hinge*_*in*_ remains unaltered by the inhibitor (Fig. 5), excluding a possible separation of the hinge loops by “β-propeller side tunnel opening”.

HDX-MS data are compatible with a “Loop side opening” mechanism (Fig. 1C) as part of the substrate gating mechanism. A “Inter-domain opening” model is also compatible (Fig. 1E), provided that the amplitude of the opening is limited. Loop A is largely unstructured (Fig. 2A, left), gets rapidly protected in the presence of inhibitor (Fig. 4) but retains significant exchange-competence (Figs 3C, blue and S3). A nick in loop A, engineered to increase freedom of motions, results in elevated, while its immobilization on loop B in abolished, PREP activity (Table S2). The observed flexibility of loop A, even at the inhibited state, and the increased dynamics of loop B at its interacting site with loop A (Fig. 2A), lend support to the kinetic studies^17^. However, PREP dynamics exclude a gating mechanism in which loop A has the exclusive role either alone or in combination with the β-propeller pore dilation (Fig. 1D).

In addition to loop A, several inter-domain loops comprise the most labile elements in the free enzyme (e.g. loop B, the His-loop and its succeeding helix, the blade 1 loop at the interface with the His-loop helical segment; Fig. 2). Blade 2 and its intra- and inter-blade-connecting loops, like D, also exhibit significant dynamics. Mutations in these flexible loops alter catalysis (Table S2) and are therefore important for PREP activity.

The stabilized inter-domain interface covers the region that extends from the α-helix connected to the His-loop to the helix-turn-helix containing Glu512 (Figs 4 and S5) of the α/β hydrolase domain. This region could potentially constitute the interfacial opening or loop rearrangement frame, though possibly without extensive separation compatible with the Glu512-Pro309 H-bond (Fig. S6A) remaining undisrupted during molecular dynamics simulations^17^. The inter-domain breathing observed by HDX may be essential for the activity of PREP, as indirectly suggested by its inactivation with disulfide cross-linking of loop B with Cys255 of the β-propeller blade 4 loop (Table S2). The limited inter-domain opening could also explain how PREP may filter substrates excluding large clients^8^.

Apart from the inhibitor-interfacing loop B, the most prominent inhibitor-induced conformational change is impacted on the His-loop, and its helical segment, and on blade 1 loop (Fig. 4). Direct inhibitor binding to the His-loop, one of the rapidly stabilized regions (Fig. 4), might rigidify the succeeding helix (that is labile in the free state; Fig. 3A, compare black and blue), thus promoting stable hydrogen bonds with and stability of its associated blade 1 loop that is distant to the active site, (Fig. S6). This may be crucial for inter-domain stability.

The induced stability in blade 1 loop is more likely the basis of *hinge*_*out*_ stabilization. *Hinge*_*out*_ (Fig. 5C) engages two backbone amides in hydrogen bonds with the blade 1 loop and the β1 strand of blade 1 at the inhibited state, based on the crystal structure (Lys75-Asn91 and Ser77-Phe89, angles and distances close to β-strand hydrogen bonds). The maximum protection observed for the blade 1 loop (Table S1; 100 sec) coincides with the onset of protection for *hinge*_out_. Although the HDX data cannot provide direct evidence of this interaction, it is likely that the His-loop, and its helical segment contribute to the stabilization of the blade 1 loop, which concomitantly could stabilize the *hinge*_*out*_.

A reversed allosteric cascade could explain the inactivation of PREP by disulfide cross-linking of blades 1 and 7 (Table S2)^8^, by locking an inter-domain closed state, rather than direct hindrance of the substrate gate (“β-propeller side tunnel opening” model). Specifically, immobilization of blade 1 could stabilize *hinge*_*out*_ and the blade 1 loop and lock the inter-domain interface through its interaction with the His-loop helix, initiating a stabilization cascade of the other interconnected flexible loops (e.g. Fig. S6).

In conclusion, inhibitor binding stabilizes a network of loops, including the His-loop^10, 19^, that are located on the front side of PREP. We now show that the previously unsuspected, His-loop connected α-helix and the blade 1 loop, are major regulatory elements involved in inter-domain closure that stabilizes the *hinge*_*out*_. Whether an induced fit mechanism^10^ or conformational selection^12^ is involved in gating cannot be concluded from our findings. The high content of fast-exchanging inter-domain loops results in poor reporting of transient closed states in the free enzyme. Nevertheless, these findings raise testable hypotheses for the elucidation of the exact role of the multiple elements at the inter-domain interface of human PREP that facilitate its gating and molecular function as a protease and possibly modulate its protein-protein interaction networks.

The structural dynamics presented here also lays the foundation for the analysis of the non-proteolytic role of PREP in neuropathology. HDX-MS is a particularly appropriate tool for these analyses given that these associations seem to involve disordered proteins like α-synuclein^5^, not easily dissected by crystallography.

## Materials and Methods

### Materials

The PREP inhibitor KYP-2047 (Fig. S1B) was synthesized as described^34^. The chromogenic substrate Z-Gly-Pro-*p*-nitroanilide was purchased from Bachem (Bubendorf, Switzerland). DTT (Dithiothreitol Biochemica) was from Applichem (Germany) and all other buffer components were of the highest quality for biochemical use from different suppliers. For HDX-MS, HEPES (for Molecular Biology) was from Fischer Scientific (USA) and DMSO (Dimethyl sulfoxide, SeccoSolve) from Merck (Germany). Guanidine hydrochloride (≥98% pure) was from Carl Roth (Germany). Deuterium oxide (D_2_O, 99.9% atom D) was purchased from Euriso-top (France), guanidine-d5 deuteriochloride (98% atom D) from Sigma-Aldrich (USA), DCl (99.5% atom D, 20% DCl in D_2_O) from Cambridge Isotope Laboratories (USA), Formic acid (Ultra-pure) from Merck KGaA (Germany) and Acetonitrile (Optima LC/MS grade) from Fischer Scientific (UK).

### Expression and purification of human PREP

A plasmid containing the human PREP cDNA (pOT7_hPREP vector, IMAGE: 3614248) was obtained from GE Dharmacon (Diegem, Belgium). The coding sequence was PCR-cloned in pET-46 Ek/LIC (Novagene) with an N-terminal hexahistidine tag using standard techniques. Human PREP was synthesized in BL21(DE3) cells and purified using immobilized Co^2+^-chelating chromatography (GE healthcare) followed by anion-exchange chromatography on a 1 ml Mono-Q column (GE healthcare). PREP activity measurements and SDS-PAGE were used to evaluate sample quality and purity. PREP activity was measured as described using the chromogenic substrate Z-Gly-Pro-p-nitroanilide (0.25 mmol/l) at pH 7.5 at 37 °C in 100 mM potassium phosphate buffer pH 7.4, 1 mM DTT^5^. The specific activity typically was 20 U/mg. One unit is defined as the amount of enzyme converting one µmole of substrate per minute in defined conditions. PREP concentration was determined from its absorbance at 280 nm using the theoretical extinction coefficient of 127090 M^−1^ cm^−1^ and by Bradford’s assay.

### HDX-MS

*Workflow*. PREP (1.5 μl, 40 μM in 50 mM HEPES-KOH pH 7.4, 50 mM KCl buffer supplemented with 1 mM DTT) was incubated at 25 °C for 30 min in the presence or absence of inhibitor. Inhibitor (0.5 μl, 1.6 mM in 50 mM HEPES-KOH pH 7.4, 50 mM KCl buffer; 16% DMSO) was added to PREP in 13-fold molar excess (800 pmol inhibitor, 60 pmol PREP) to ensure the complete inhibitor-bound state. For the free state, 0.5 μl of 16% DMSO 50 mM HEPES-KOH pH 7.4, 50 mM KCl buffer was added to PREP during incubation at 25 °C, to a final 4% DMSO, for identical conditions with the inhibitor-bound state. At 30 min of incubation, PREP was isotopically labeled with 95.29% final D content at 25 °C by addition of 40.5 μl deuterated buffer (lyophilized HEPES-KOH pD 7.4, 50 mM KCl buffer freshly re-suspended in D_2_O and supplemented with 1 mM DTT) for time intervals of 10, 30, 100, 1,000 and 10,000 sec. pD refers to the corrected value for the isotope effect. The HDX reaction was quenched at the defined time intervals by instant acidification (pD 2.5; formic acid) and snap freezing in liquid nitrogen. The pre-chilled quenching solution contained guanidine hydrochloride and DTT to a final concentration of 2 M and 20 mM respectively, to increase the peptide coverage by mild PREP denaturation and reduction. In order to examine the instant inhibitor effect on PREP at the initial binding step, inhibitor was added to PREP (13 fold molar excess) only during isotope labeling for 10 sec, in an individual experiment. The obtained results were similar to the pre-incubated state and therefore it was not tested for other time intervals. Undeuterated PREP was treated as above but in protiated buffer for peptide identification. For the full deuteration control, PREP (1 μl, 119.6 μM) was incubated in deuterated buffer (as described above) containing 6 M guanidine-d5 deuteriochloride (95.29% final D content) at 25 °C for 3 h, in the presence and absence of inhibitor (DMSO was taken into account as above). The addition of inhibitor in the specific control accounted for the observed PREP autocleavage under the denaturing conditions that led to loss of specific peptides containing prolyl and alanyl residues. The full deuteration reaction was quenched to pD 2.5, 2 M guanidine-d5 deuteriochloride, 20 mM DTT and instantly frozen in liquid nitrogen.

#### Online proteolysis-LC-MS analysis

The quenched samples were instantly thawed by spinning and injected (50 μl injection loop; 50 pmol PREP injected) into a nanoACQUITY UPLC System with HDX technology (Waters, UK), thermostated at the digestion and LC separation chambers at 20 °C and 0.2 °C respectively. Proteolytic digestion (Enzymate BEH pepsin column, Waters) and peptide trapping/desalting (ACQUITY UPLC R BEH C18 VanGuard pre-column; 130 Å, 1.7 μm, 2.1 × 5 mm; Waters) were performed with 0.23% formic acid in H_2_O (Solvent A) at 100 μl/min for 3 min, online with peptic peptide separation (ACQUITY UPLC R BEH C18 analytical column; 130 Å, 1.7 μm, 1 × 100 mm; Waters) at 40 μl/min using a 17 min linear gradient from 5 to 50% Solvent B (ACN, 0.23% formic acid). At 18 min Solvent B was set to 90%. The eluate was analyzed online on a Synapt G2 ESI-Q-TOF instrument (Waters, UK) with a MassLynX interface (version 4.1 SCN870; Waters) for data collection. The source/TOF conditions were set as: resolution mode, capillary voltage 3.0 kV, sampling cone voltage 20 V, extraction cone voltage 5 V, source temperature 80 °C, desolvation gas flow 500 L/h at 150 °C. The deuterated samples were analyzed in MS acquisition mode, while the undeuterated sample in MS^E^ acquisition mode over the m/z range 100–2,000 Da, using a collision energy ramp from 15 to 35 V. Leucine Enkephalin (2 ng/μl in 50% ACN, 0.1% formic acid; 5 μl/min) was co-infused in both acquisition modes for accurate mass measurements (reference mas: m/z 556.2771).

#### Data analysis and statistical aspects

For peptide identification, MS^E^ data were processed on the ProteinLynx Global Server (PLGS v3.0.1, Waters, UK), using a user-defined database containing the PREP sequence under the following criteria: digestion enzyme, non-specific; false discovery rate, 4%, minimum fragment ion matches/peptide and/protein, 3 and 7; minimum peptide matches/protein, 1; low and elevated energy thresholds, 150 and 25 counts; intensity threshold: 500 counts, reference mass correction window, 0.25 Da at 556.2771 Da/e. The identified peptides from two independent MS^E^ raw files were further filtered on the DynamX software (version 3.0, Waters) for DynamX score >7, maximum MH + error of 5 ppm and minimum products/amino acid of 0.2. Only robustly identified peptides in both replicates were further processed, resulting in 91.8% PREP sequence coverage (Fig. S2). It must be noted that the peptide containing the active site residue Ser554 (peptic fragment 536–559) was identified and observed in all free-state samples but not in the inhibitor-bound state, due to the covalent interaction of Ser554 with the inhibitor. All HDX reactions were performed in triplicates. In most peptic fragments of the inter-domain loop regions discussed in the results, the loop content corresponds to at least ^3^/_5_ of the peptic fragment (e.g. blade 5 loop; peptide 301–311; ^3^/_4_ loop, ^1^/_4_ preceding β-strand of the total exchangeable NHs). An exception of high loop content in peptic fragments corresponds to the blade 2 loop (peptide 139–149; Fig. S2) that exhibits only ^2^/_5_ loop content. For visualization of the PREP dynamics, deuterium uptake values were determined relatively to the maximum uptake acquired by the full deuteration control, as $\% D=(\frac{{m}_{HDX}-{m}_{UNL}}{{m}_{FD}-{m}_{UNL}})\times 100$, where *m* corresponds to the measured centroid mass of the deuterated sample (*m*_*HDX*_), the reference undeuterated sample (*m*_*UNL*_) and the full deuteration control (*m*_*FD*_). Statistical analysis of the significance of differences in the free and inhibitor-bound state was achieved using a modified approach of Bennett *et al*.^35, 36^. Briefly, two-tail paired t-tests, comparing the mean uptake, as absolute deuterium uptake values, of the two states for each peptide, were performed using R language and the significance threshold was set to 95% confidence (1−p > 0.95). An additional threshold was set at ± 4 SD of average pooled standard deviations of both states. Finally, to exclude false positives due to high SD outliers that are averaged out in the pooled SDs, we introduced the following criterion. The difference between the two states for each peptide must exceed twice the sum of SDs of the two states for the given peptide. Only differences that fulfill all three criteria were considered as statistically significant. Visualization of the statistical analysis in scatter plots was achieved by R language.

## Electronic supplementary material

Supplementary Material

Table S1
