# Supplementary material for: Dynamics and ligand-induced conformational changes in human prolyl oligopeptidase analyzed by hydrogen/deuterium exchange mass spectrometry
Source: Sci Rep. 2017 May 26;7:2456. doi: 10.1038/s41598-017-02550-1 (PMC5446394; doi:10.1038/s41598-017-02550-1)
Supplement: Supplementary file 1 — Supplementary Material [file 41598_2017_2550_MOESM1_ESM.pdf]

SUPPLEMENTARY INFORMATION

Dynamics and ligand-induced conformational changes in human prolyl oligopeptidase analyzed by hydrogen/deuterium exchange mass spectrometry

Alexandra Tsirigotaki[1], Roos Van Elzen[2], Pieter Van Der Veken[3], Anne-Marie Lambeir[2] and Anastassios Economou[1].

Table of contents

Supplementary figures

Figure S1: Effects of the inhibitor KYP-2047 on the structure, binding kinetics and surface-accessibility of human PREP (related to Figures 2, 4 and 5).

Figure S2: Peptide coverage and detailed deuterium uptake heat map shown on the sequence of human PREP at the free state (related to Figure 2).

Figure S3: Peptide coverage and detailed deuterium uptake heat map on the sequence of human PREP at the inhibitor-bound state (related to Figures 4 and 5).

Figure S4: Deuterium uptake difference plot of free and inhibitor-bound human PREP (related to Figures 4 and 5).

Figure S5: Inhibitor interfacing residues in the human PREP structure (related to Figures 4, 5).

Figure S6: Inter- and intra-domain hydrogen bond network in the human PREP structure (related to Figures 2, 4, 5).

Supplementary tables

Table S1: Deuterium uptake values and statistical analysis on the free and inhibitor-bound human PREP (related to Figures 2, 4, 5).

Table S2: Characterized site-directed mutants of PREP and their impact on structure and activity.

Supplementary Materials and Methods

- Native page
- Inhibitor binding kinetics
- Titration of free sulfhydryl groups

Supplementary References

Supplementary figures

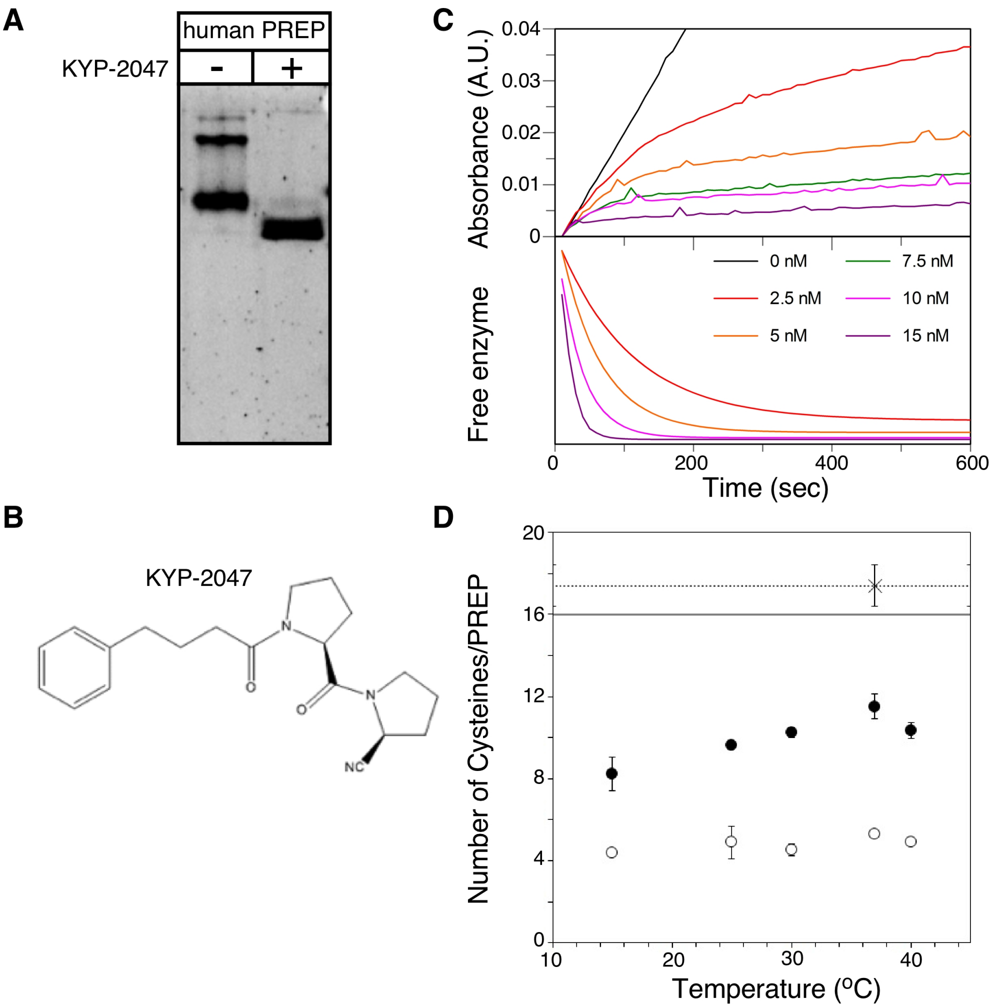

Figure S1, Effects of the inhibitor KYP-2047 on the structure, binding kinetics and surface-accessibility of human PREP (related to Figures 2, 4 and 5)

A. Native-PAGE of human PREP (8 μl of 1.5 μM in 0.1 M Tris pH 7.4, 3 mM DTT) in the absence and presence of the inhibitor KYP-2047 (12 μM). While the human PREP at the free state (left) migrates as a collection of bands, the inhibitor-treated PREP (right) migrates faster as a single band.

B. The structure of the PREP inhibitor used in this study. KYP-2047 is a prototypical PREP inhibitor based on a Pro-pyrrolidine scaffold containing a cyano-group targeting the oxyanion hole[1]. In the crystal structure (PDB accession entry 4AN0) the cyano carbon atom covalently interacts with the catalytic Ser554[2].

C. Kinetic analysis of inhibitor binding. The top panel displays absorbance progress curves after mixing PREP (1 nM) with different concentrations of inhibitor (as indicated) in the presence of 0.5 mM Suc-Ala-Pro-pNA at 37°C. Initial rate (v_i) and steady-state rate (v_s) are obtained as the tangents of the curve at time 0 and at the end of the reaction. The time to

change from v_i to v_s is used to calculate the pseudo-first order rate constant (k_{obs}) of inhibitor binding. The k_{obs} has an hyperbolic dependency on the inhibitor concentration, indicative of the existence of a pre-equilibrium (with K_1 = 10 nM) followed by a slow first order reaction ($k_2 \geq 0.05\ s^{-1}$) as published[3]. The lower panel shows the time course by which the free enzyme disappears as a result of inhibitor binding, calculated after fitting the progress curves with the following equation: $A = (v_o - v_s)*(1 - \exp(-k_{obs}*t))/k_{obs} + v_s*t$ (A is the p-nitroaniline absorbance at 405 nm and t is time). These results mean that at an inhibitor concentration of 800 μM, PREP is fully saturated with inhibitor and it takes about 60 s to form the high-affinity complex.

D. Temperature- and inhibitor binding-dependent differences in surface accessibility of cysteines in PREP. PREP contains 16 cysteines (filled line), of which 8-12 are readily accessible in the free enzyme under native conditions in a temperature-dependent manner (filled circles), and 16 in denaturing conditions (upper section, dashed line). In the presence of the inhibitor (open circles) labelling of only 4 cysteines is observed at all temperatures tested, suggesting that PREP acquires a more compact structural state. The data represent the average and standard deviation of two independent measurements.

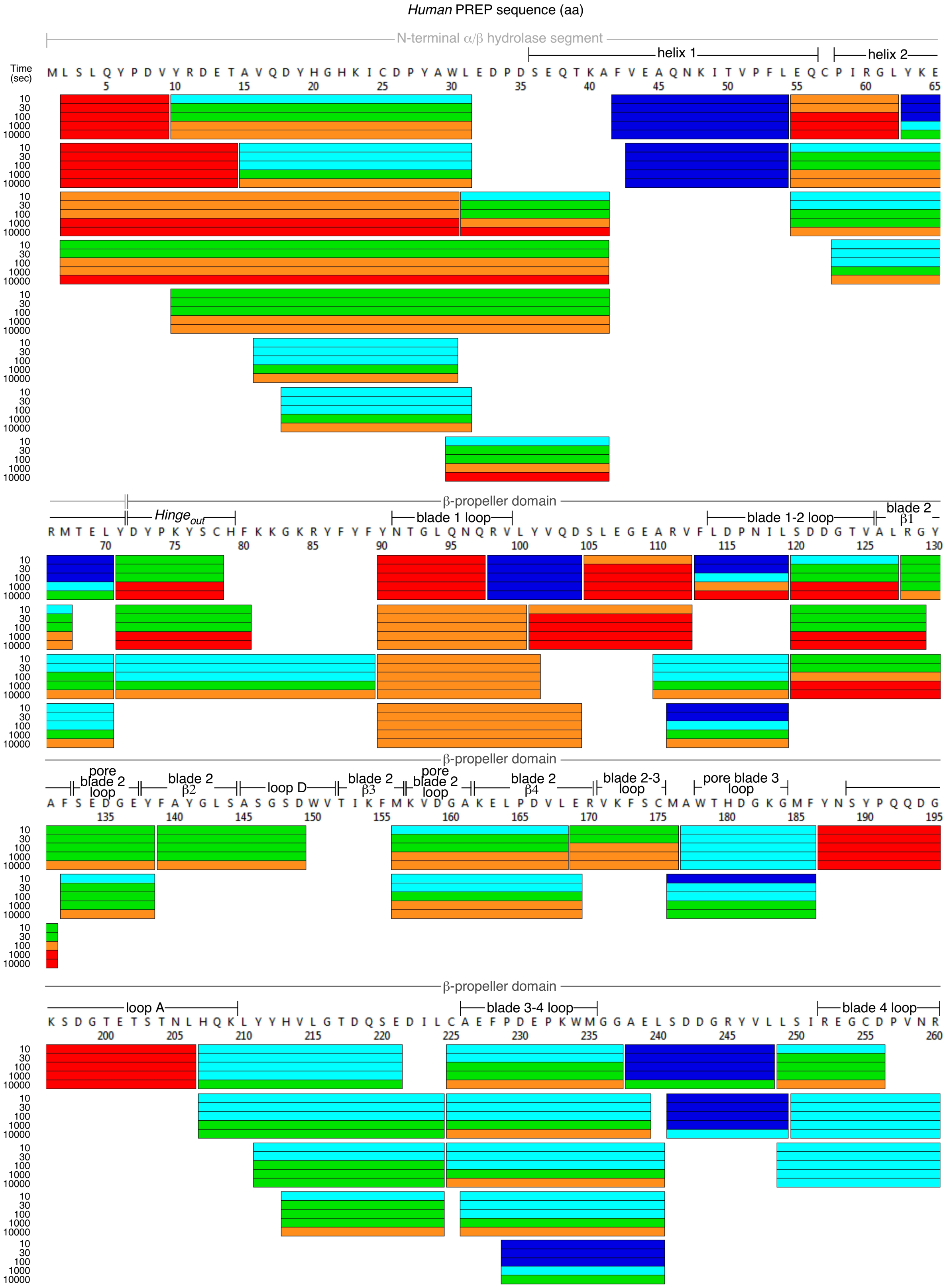

Continues on next page

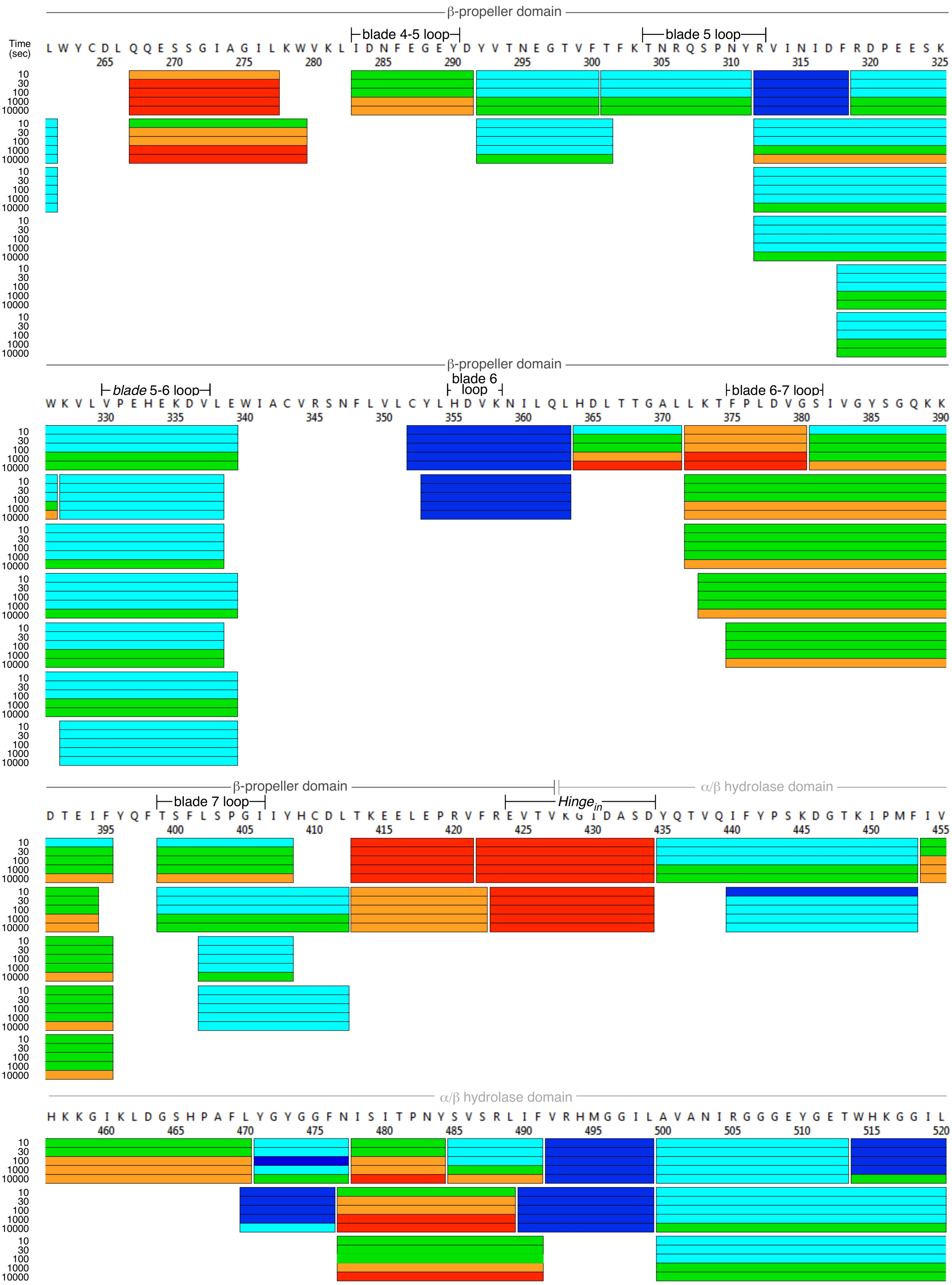

Continues on next page

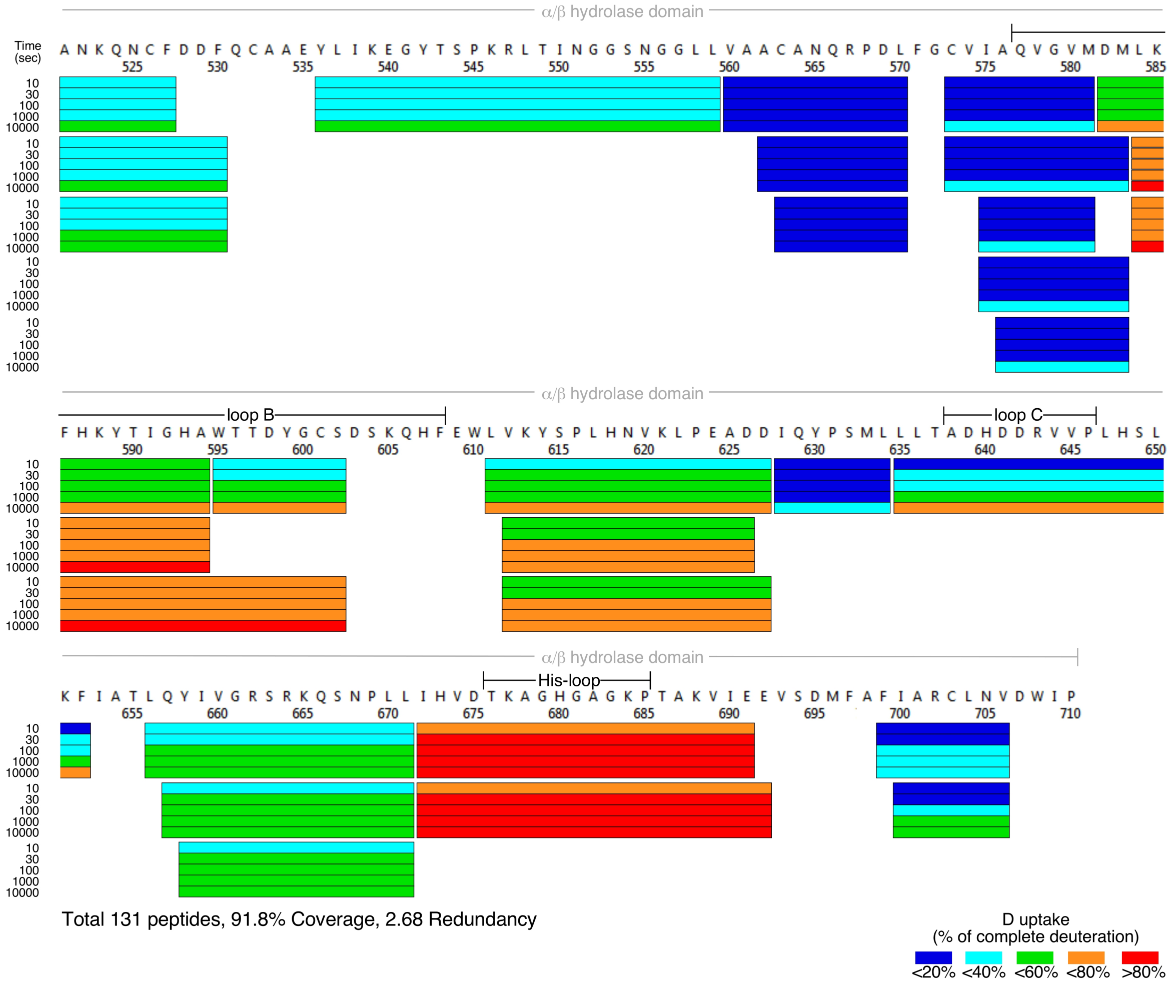

Figure S2, Peptide coverage and detailed deuterium uptake heat map on the sequence of human PREP at the free state (related to Figure 2)

Deuterium uptake values of human PREP peptic peptides at the free state, relatively to the complete deuteration control, are mapped on the primary structure using a color gradient (see bottom right). The identified peptides, represented by space-separated bars, result in 91.8% coverage of the sequence. Vertically stacked bars of the same length correspond to deuterium uptake values of the same peptide at increasing H/D exchange pulses, as indicated (left) (numerical values in Table S1). Elements discussed in the text are assigned (on top of the corresponding sequence; inter-domain loops with no common nomenclature are represented by the number of the blade in which they reside; inter-blade connecting loops at the inter-domain interface are separated with dash; loops facing inwards the β-propeller pore are indicated as pore loops; β-propeller β-strands are enumerated in hierarchical order within the indicated blade). *n=3*

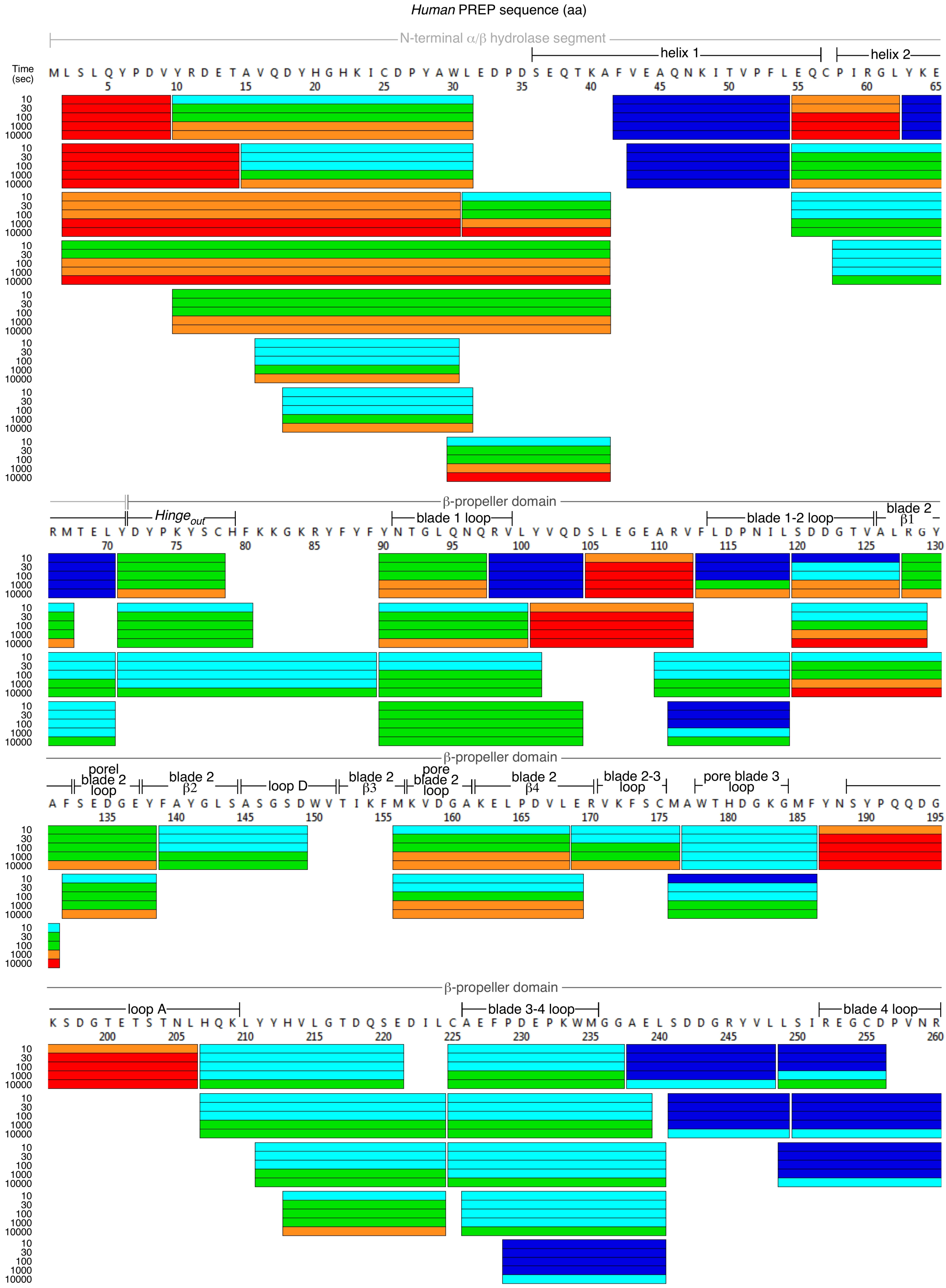

Continues on next page

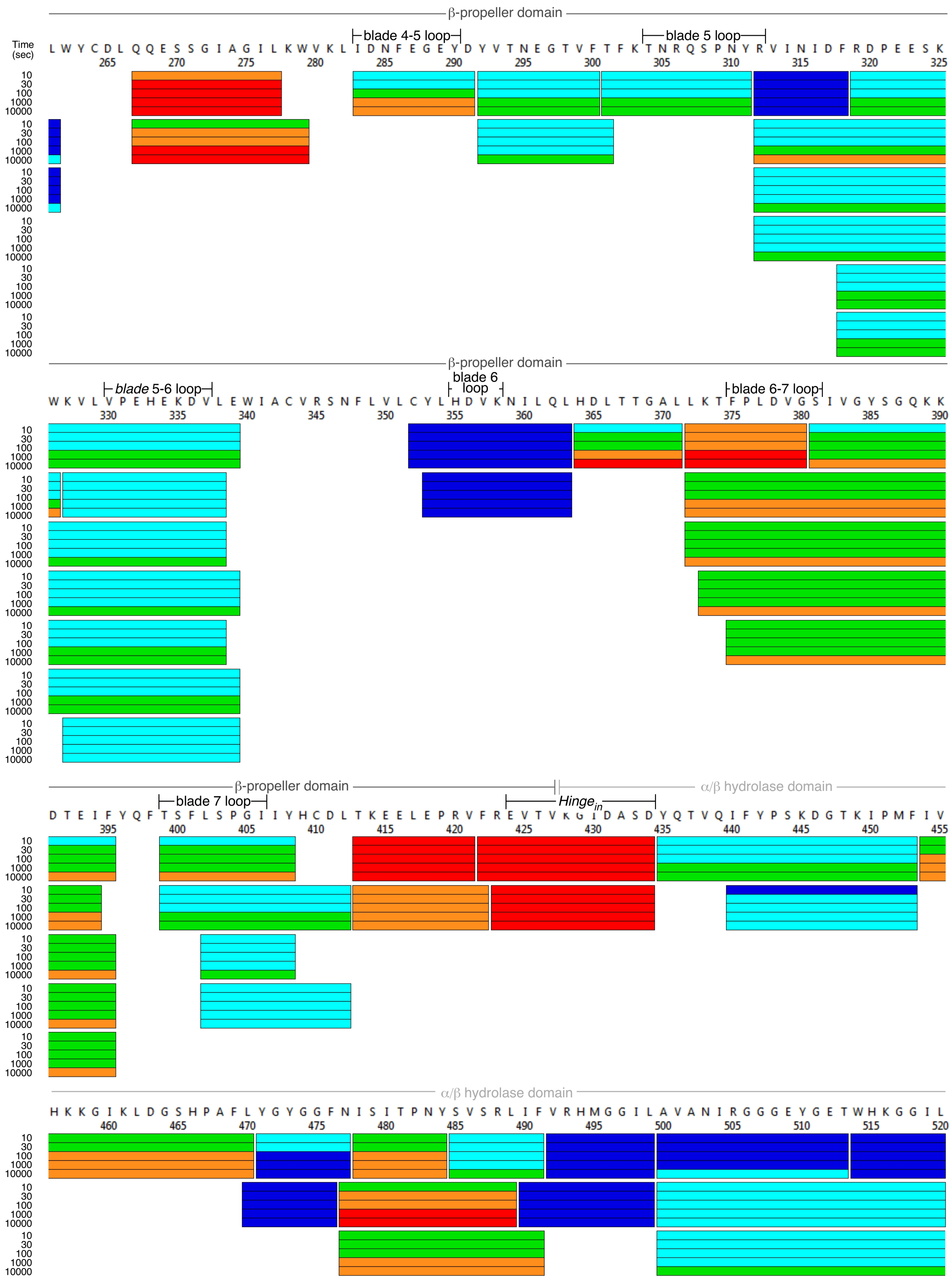

Continues on next page

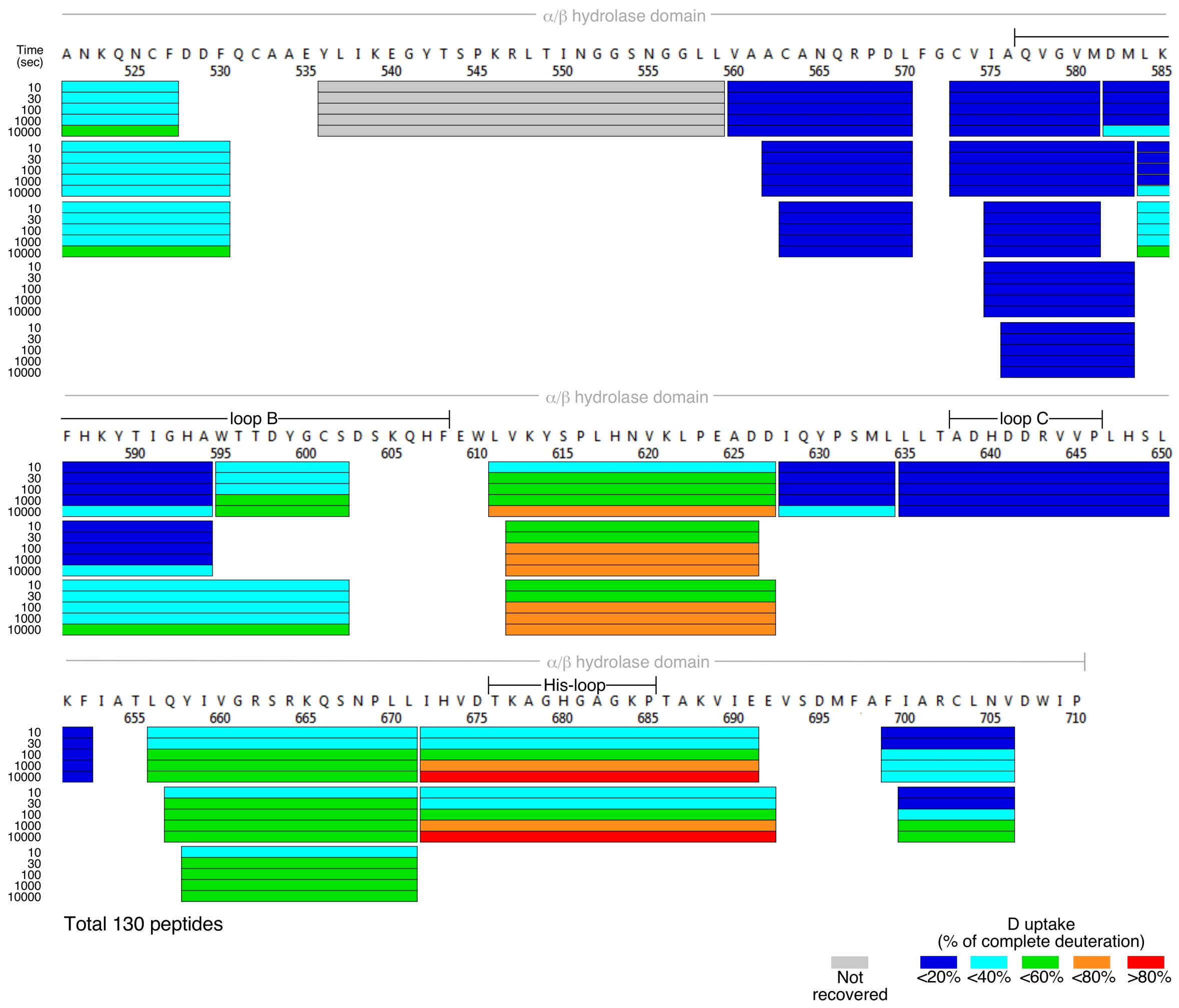

Figure S3, Peptide coverage and detailed deuterium uptake heat map on the sequence of human PREP at the inhibitor-bound state (related to Figures 4 and 5)

Deuterium uptake values of human PREP peptic peptides at the inhibitor-bound state, relatively to the complete deuteration control, are mapped on the primary structure using a color gradient (bottom right), as in Figure S2 (numerical values in Table S1). The peptide 536-559 (highlighted in grey), containing Ser554 of the active site, was not recovered at this state due to its covalent interaction with the inhibitor, and was excluded from direct comparison with the free-state. Elements discussed in the text are assigned (as in Fig. S2). *n=3*. Description of the overlapping peptides-driven localization of inhibitor-induced protection at Ile478, and Asn477: The peptides with residues 477-489 and 477-491 exhibit reduced D uptake in the inhibitor-bound state resolved already at short deuteration pulses (t<1 min), while fast protection is not observed for peptide 485-491, that covers a helix distant from the active site, neither for peptides 471-477 and 478-484. Apparently, the affected residues lie in the early residues of peptides 477-489 and 477-491. Deuterium incorporation in N-terminal residues in deuterated peptides is unavoidably back-exchanged to

H during analysis[4,5], an effect that is also observed for C-terminal residues[5,6]. From the combined analysis of these overlapping peptides, it appears that Ile478, and perhaps Asn477, if C-terminal back-exchange has occurred in peptide 471-477, are the residues most rapidly stabilized by the inhibitor. In contrast, the long loop segment at the inter-domain interface (residues 479-484) is unaffected by the inhibitor throughout the H/D exchange time course (see also Table S1).

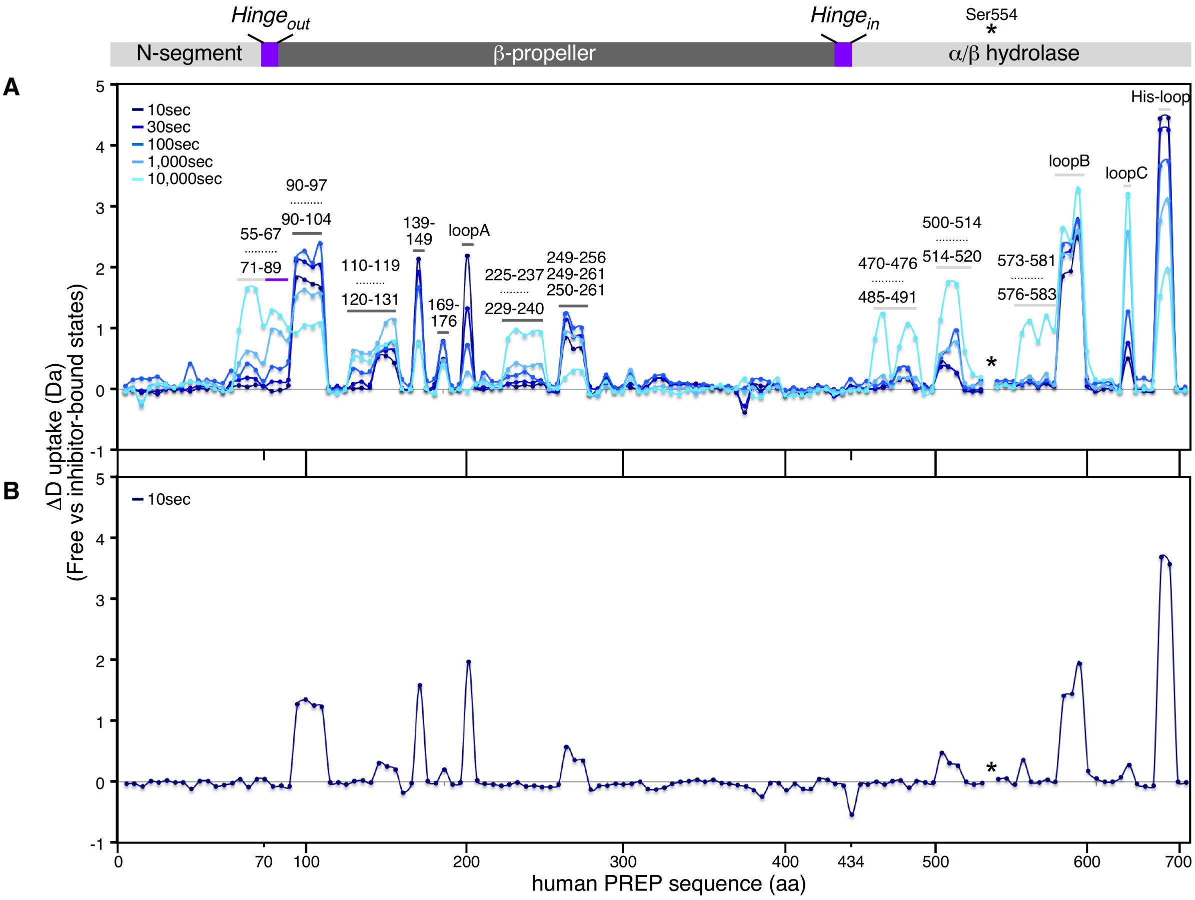

Figure S4, Deuterium uptake difference plot of free and inhibitor-bound human PREP (related to Figures 4 and 5)

A-B. The absolute deuterium uptake difference between the free and inhibitor-bound states of human PREP is given for all identified peptides (indicated by dots; overlapping peptides are given in increasing order of first residue position on the sequence) along the PREP sequence (x axis: approximate residue position on the primary sequence). The structural domains of PREP are indicated (top; colored as in Figure 1B) in alignment with the corresponding peptides (A-B) covering each domain. Peptides that take up less D in the presence of bound inhibitor are annotated according to commonly used nomenclature where available (Panel A; e.g. loop A). Peptides with no specific nomenclature are labeled by their first and last residue numbers. For protected regions covered by more than one peptide only the first and last peptides are indicated separated by dots, e.g. 90-97······90-104. The peptide containing the active site Ser554 (indicated by an asterisk) was not recovered in the inhibitor-bound state due to their covalent interaction, and is excluded from comparison.

A. D uptake difference plot of free and inhibitor-bound PREP, monitored at a steady state (pre-incubation with inhibitor prior to H/D exchange). Values at increasing isotope labeling pulses are assigned with different colors (as indicated, upper left). *n=3*

B. D uptake difference plot of free and inhibitor-bound PREP, derived from a 10 sec pulse during which inhibitor addition and D uptake occurred simultaneously. At the molar excess of inhibitor used, inhibitor binding by PREP is saturated rapidly. *n=2*

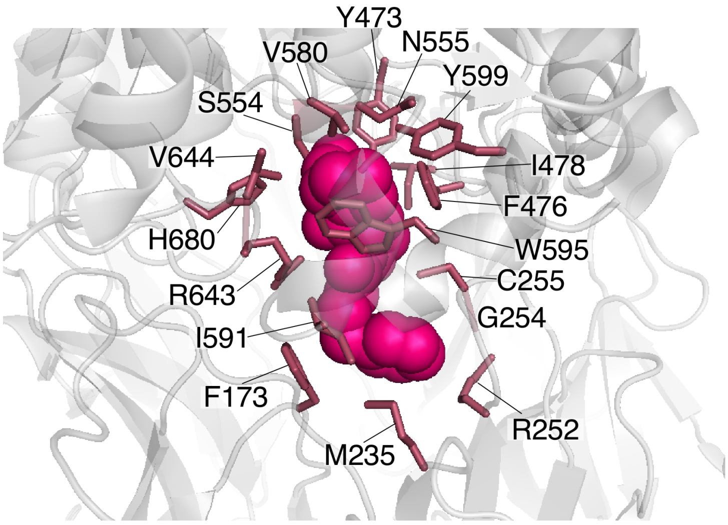

Figure S5, Inhibitor interfacing residues in the human PREP structure (related to Figures 4, 5)

Map of inhibitor-interfacing residues on the structure of human PREP (PDB accession entry 3DDU; side chains of interfacing residues shown as sticks; dark red). The inhibitor KYP-2047 (magenta; spheres) was positioned by alignment of the human PREP structure with the inhibitor-bound porcine PREP structure (PDB accession entry 4AN0). The inhibitor-interfacing residues were identified by the PDBePISA server (v1.51; http://www.ebi.ac.uk/msd-srv/prot_int/cgi-bin/piserver) using the 4AN0 structure, and were confirmed for identical side chain orientation in the human PREP structure. Inhibitor-interfacing residues that are rapidly protected by the inhibitor, as seen by HDX-MS (Fig. 4) include Arg643, Val644 of loop C, His680 of the His-loop, Phe173 of the blade 2-3 loop, Ile591, Trp595 of loop B, Arg252, Gly254, Cys255 of the blade 4 loop and Ile478 of the loop 477-485. Slow response to inhibitor-binding was observed (Fig. 5) for the inhibitor-interfacing residues Val580 and Tyr599 of the N- and C-terminal loop B segments respectively, Tyr473 and Phe476 of the α/β hydrolase loop 471-477 that surrounds the inhibitor and Met235 of the blade 3-4 loop of the β-propeller domain. Ser554 and Asn555 were not included in comparative HDX-MS analysis, due to covalent interaction of Ser554 with the inhibitor (Asn555 was identified in the same peptide with Ser554 and therefore excluded).

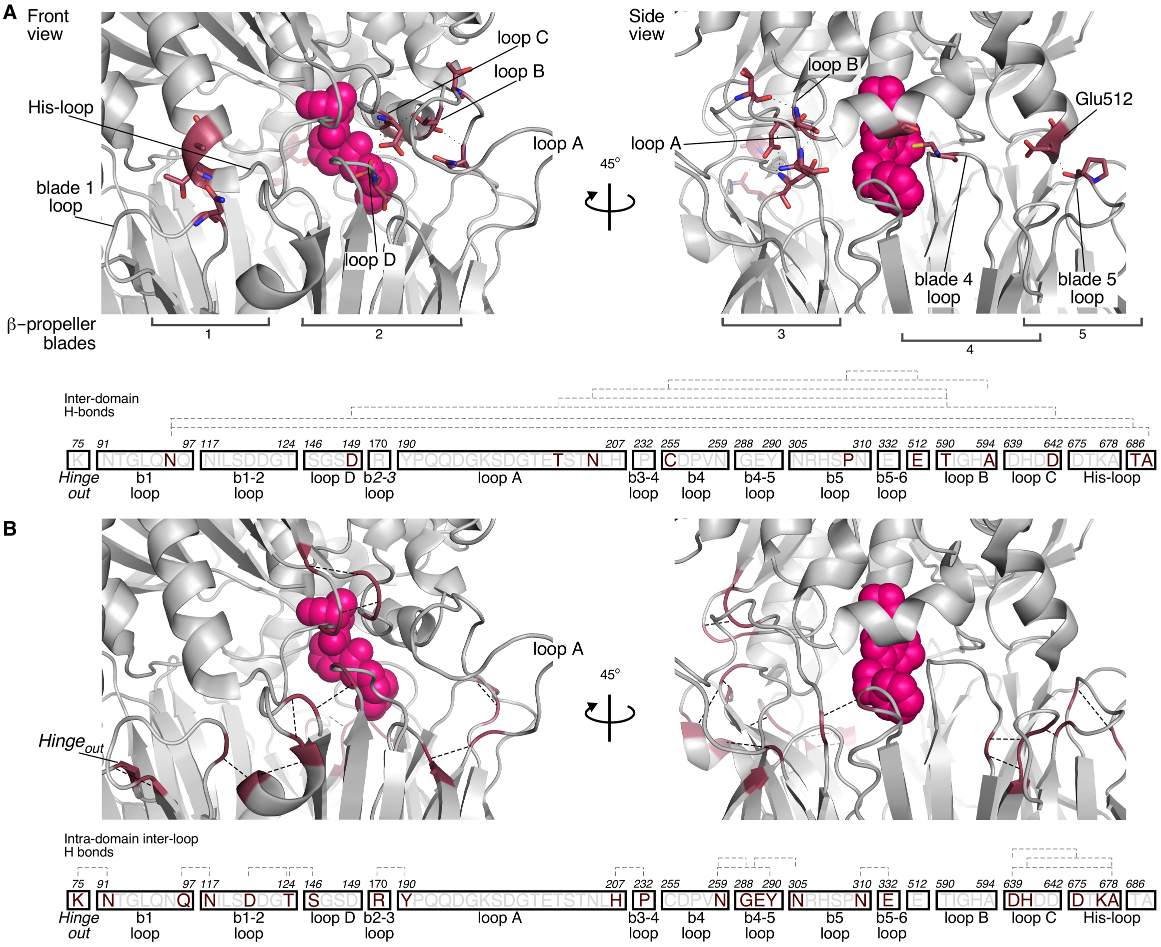

Figure S6, Inter- and intra-domain hydrogen bond network in the human PREP structure (related to Figures 2, 4 and 5)

A-B. Ribbon representation of the porcine PREP structure (grey; PDB accession entry 4AN0) at the closed state in a front (left) and side view (right). The inhibitor KYP-2047 (magenta) is shown in spheres. Residues that participate in hydrogen bonds containing backbone amides (side chain-main chain, main chain-main chain) at the inter-domain interface (panel A) or within the same domain but at inter-connecting loops (panel B) are highlighted (dark red; in panel A shown as sticks, blue, red and yellow indicate nitrogen, oxygen and sulfur atoms respectively). Hydrogen bonds are represented by dashed lines in the structures (top) and on the sequence (bottom; b indicates β-propeller blade). Loops and interacting regions are indicated and the β-propeller blades are enumerated (inter-domain loops with no common nomenclature are represented by the number of the blade in which they reside; inter-blade connecting loops are separated with dash). Hydrogen bonds were identified in the human PREP structure (PDB accession entry 3DDU) and are identical with those of the porcine PREP structure.

Supplementary tables

Table S1 Deuterium uptake values and statistical analysis on the free and inhibitor-bound human PREP (related to Figures 2, 4, 5) (xls file accessible via the following link)
https://www.dropbox.com/s/s9b1gghpoe049oi/Table%20S1.xls?dl=0

Table S2, Characterized site-directed mutants of PREP and their impact on structure and activity. PREP mutations and their effect, as reported elsewhere, are summarized together with the corresponding references.

Mutation	Rationale	Properties	Ref
T202C-T590C	Cross-linking loops A and B	40-fold decrease in rate when reduced, inactivation when the disulfide bridge is formed	[2]
Y187_K209delins TGGTQ	Removal of loop A by replacement with a short linker	10000-fold decrease in rate	[2]
K196^S197	Trypsin cleaved loop A	1.5-fold increase in rate, altered pH profile	[2]
T202C	Loop A mutant	Increased activity, increased mobility	[7]
T204A	Loop A mutant	Wild type activity	[7]
T590C	Loop B mutant	Preference for shorter substrates	[7]
H680A	Catalytic histidine	Inactivation, His-loop and loop A disordered in crystal structure	[7]
D149A	Loop D mutant	50-fold decreased activity and altered pH profile	[7]
C255T	Mutation in propeller loop that makes contact with the inhibitor	Decreased activity, pH optimum shifted to acidic form<br>Higher Km	[7]
Q397C	Disulfide cross-linking of propeller blades 1 and 7	Inactivation	[8]
T597C	C255-loop B disulfide bond	Inactivation	[9]

Supplementary Materials and Methods

Native-PAGE

Human PREP was separated on a discontinuous 10% acrylamide Native-PAGE while omitting denaturing and reducing conditions. The electrophoresis was carried out at low voltage settings (<60 V) to minimize heat generation. Before starting the electrophoresis, PREP (1.3 μM) was incubated for 15 min at 25°C with or without a ~10x molar excess of inhibitor KYP-2047 in 0.1 M Tris-HCl pH 7.4, 3 mM DTT. The proteins were visualized using Oriole fluorescent stain (Bio-Rad Laboratories).

Inhibitor binding kinetics

The kinetic constants of KYP-2047 binding to recombinant human PREP were determined as described[3].

Titration of free sulfhydryl groups

The amount of available sulfhydryl groups in PREP were determined using 5,5,-dithio-bis-(2-nitrobenzoic acid (DTNB or Ellman's reagent). After adding the reagent in a > 80 molar excess the concentration of available thiols was determined from its absorbance at 412 nm and extinction coefficient of 12907.26 $M^{-1}cm^{-1}$. The total amount of available thiol groups was determined after denaturation of PREP using 4 M Guanidine hydrochloride in 0.1 M Tris-HCl pH 7.4 or in native conditions in 0.1 M Tris-HCl pH 7.4, in presence or absence of inhibitor KYP-2047 (10x molar excess) at different temperatures. In all cases care was taken to excessively dialyze or buffer exchange (3x micro Bio-spin P6 column (Bio-Rad)) the protein samples to minimize interference with DTT.

Supplementary References

1 Wallen, E. A. *et al.* Conformationally rigid N-acyl-5-alkyl-L-prolyl-pyrrolidines as prolyl oligopeptidase inhibitors. *Bioorg Med Chem* **11**, 3611-3619 (2003).

2 Kaszuba, K. *et al.* Molecular dynamics, crystallography and mutagenesis studies on the substrate gating mechanism of prolyl oligopeptidase. *Biochimie* **94**, 1398-1411, doi:10.1016/j.biochi.2012.03.012 (2012).

3 Van Elzen, R., Schoenmakers, E., Brandt, I., Van Der Veken, P. & Lambeir, A. M. Ligand-induced conformational changes in prolyl oligopeptidase: a kinetic approach. *Protein Eng Des Sel*, doi:10.1093/protein/gzw079 (2017).

4 Walters, B. T., Ricciuti, A., Mayne, L. & Englander, S. W. Minimizing back exchange in the hydrogen exchange-mass spectrometry experiment. *J Am Soc Mass Spectrom* **23**, 2132-2139, doi:10.1007/s13361-012-0476-x (2012).

5 Bai, Y., Milne, J. S., Mayne, L. & Englander, S. W. Primary structure effects on peptide group hydrogen exchange. *Proteins* **17**, 75-86, doi:10.1002/prot.340170110 (1993).

6 Percy, A. J., Rey, M., Burns, K. M. & Schriemer, D. C. Probing protein interactions with hydrogen/deuterium exchange and mass spectrometry-a review. *Anal Chim Acta* **721**, 7-21, doi:10.1016/j.aca.2012.01.037 (2012).

7 Szeltner, Z. *et al.* The loops facing the active site of prolyl oligopeptidase are crucial components in substrate gating and specificity. *Biochim Biophys Acta* **1834**, 98-111, doi:10.1016/j.bbapap.2012.08.012 (2013).

8 Fulop, V., Szeltner, Z. & Polgar, L. Catalysis of serine oligopeptidases is controlled by a gating filter mechanism. *EMBO Rep* **1**, 277-281, doi:10.1093/embo-reports/kvd048 (2000).

9 Szeltner, Z. *et al.* Concerted structural changes in the peptidase and the propeller domains of prolyl oligopeptidase are required for substrate binding. *J Mol Biol* **340**, 627-637, doi:10.1016/j.jmb.2004.05.011 (2004).
